# BCG priming followed by a novel interleukin combination activates Natural Killer cells to selectively proliferate and become anti-tumour long-lived effectors

**DOI:** 10.1038/s41598-024-62968-2

**Published:** 2024-06-07

**Authors:** María-José Felgueres, Gloria Esteso, Álvaro F. García-Jiménez, Ana Dopazo, Nacho Aguiló, Carmen Mestre-Durán, Luis Martínez-Piñeiro, Antonio Pérez-Martínez, Hugh T. Reyburn, Mar Valés-Gómez

**Affiliations:** 1 Department of Immunology and Oncology, National Centre for Biotechnology, Spanish National Research Council (CNB-CSIC), Darwin, 3, 28049 Madrid, Spain; 2 Genomics Unit, Centro Nacional de Investigaciones Cardiovasculares (CNIC), Madrid, Spain; 3 CIBER de Enfermedades Cardiovasculares (CIBERCV), Madrid, Spain; 4 Department of Microbiology, Pediatrics, Radiology and Public Health of the University of Zaragoza, IIS Aragon, CIBER de Enfermedades Respiratorias, Zaragoza, Spain; 5 Translational Research in Pediatric Oncology, Hematopoietic Transplantation and Cell Therapy, IdiPAZ, and Pediatric Hemato-Oncology, Hospital Universitario La Paz, Madrid, Spain; 6 IdiPAZ-CNIO Pediatric Onco-Hematology Clinical Research Unit, Spanish National Cancer Research Centre (CNIO), 28049 Madrid, Spain; 7 Urology Department and Hospital La Paz Institute for Health Research (IdiPAZ), La Paz University Hospital, Madrid, Spain; 8 Pediatric Department, Autonomous University of Madrid, Madrid, Spain

**Keywords:** Cancer immunology, Cell immunotherapy, NK cells, BCG, Cytokine activation, Biological techniques, Immunology

## Abstract

The short-lived nature and heterogeneity of Natural Killer (NK) cells limit the development of NK cell-based therapies, despite their proven safety and efficacy against cancer. Here, we describe the biological basis, detailed phenotype and function of long-lived anti-tumour human NK cells (CD56^high^CD16^+^), obtained without cell sorting or feeder cells, after priming of peripheral blood cells with *Bacillus Calmette-Guérin* (BCG). Further, we demonstrate that survival doses of a cytokine combination, excluding IL18, administered just weekly to BCG-primed NK cells avoids innate lymphocyte exhaustion and leads to specific long-term proliferation of innate cells that exert potent cytotoxic function against a broad range of solid tumours, mainly through NKG2D. Strikingly, a NKG2C^+^CD57^-^FcεRIγ^+^ NK cell population expands after BCG and cytokine stimulation, independently of HCMV serology. This strategy was exploited to rescue anti-tumour NK cells even from the suppressor environment of cancer patients’ bone marrow, demonstrating that BCG confers durable anti-tumour features to NK cells.

## Introduction

Natural killer (NK) cells are cytotoxic innate immune lymphocytes with potent anti-tumour capacities that make them potential efficient agents for cancer immunotherapy^1–5^. Specifically, cytotoxic tumour recognition in the absence of adverse events like cytokine release syndrome, neurotoxicity, or graft-versus-host disease (GVHD)^6^ make NK cells very attractive candidates for universal “off-the-shelf” cell therapies. So, new protocols for ex vivo NK cell priming and expansion, using different cell sources and enhancers of effector functions are currently being tested for clinical use.

Importantly, NK cells are an extremely heterogeneous population^7,8^ and only certain subsets are active for tumour elimination. In peripheral blood, the majority of circulating human NK cells have low-density of CD56. This CD56^dim^ population, considered the mature cytotoxic NK cell subset, usually expresses high levels of CD16 (FcγRIII) and can also mediate antibody-dependent cellular cytotoxicity (ADCC)^9^. In contrast, CD56^bright^ NK cells are generally less cytotoxic, express little or no CD16, and are considered regulatory. CD56^dim^ and CD56^bright^ NK cells express different levels of activating and inhibitory receptors including KIRs, CD94/NKG2 heterodimers, and NCRs among others, contributing to the balance of signals that regulate NK function [for review^10^]. This heterogeneity of NK cell phenotypes results in a very versatile immune cell type that can mediate a large variety of specific functions, including the elimination of pathogen-infected and cancer cells, but generally, specific NK subpopulations are is present in relatively low frequency.

Studies of NK cell education and differentiation in secondary lymphoid tissues revealed the strong influence exerted by cytokines, such as IL15, IL12, IL18, IL21 and IFNα/β on NK maturation and function^11–13^. In vitro use of these cytokines has allowed characterization of distinct phenotypes and functions of NK cells. IL12 alone sustains NK cell viability without proliferation and acts synergistically with IL18 and IL21 to stimulate IFNγ production^14–16^. IL15 is essential for both NK differentiation and survival and considerably enhances the anti-tumour response of activated NK cells^17^. So, brief exposure of NK cells to high doses of IL12, IL15 and IL18 results in the upregulation of IFNγ, perforin and granzymes^18^. These cytokine-induced memory-like (CIML) NK cells^19,20^, which produce IFNγ in response to tumour cells upon cytokine re-stimulation, have shown promising results against haematological tumours when tested in clinical trials^21^, but unfortunately many of these remissions last only a few months, presumably because of the short-lived nature of the transferred NK cells.

Direct intravesical instillations of *Bacillus Calmette-Guérin* (BCG) is one of the first successful cancer immunotherapies, used for decades for the treatment of non-muscle invasive bladder cancer (NMIBC) with nearly 70% tumour-free survival rate^22^. BCG-primed NK cells (**B-pNK**) upregulate CD56, kill bladder cancer cells efficiently and express CD16, KIRs, CD57 and CD94/NKG2A^23^, a phenotype consistent with an anti-tumour CD56^high^CD16^+^ phenotype previously described for cytokine-activated NK cells^24^ and reminiscent of CIML-NK cells^20^. Whether anti-tumour B-pNK cells have features of CIML, other adaptive-like^25,26^ or trained-immunity activity^27,28^ is still unknown. We demonstrated previously that very low levels of soluble factors released by peripheral blood mononuclear cells (PBMC) in the context of BCG were a crucial step for NK cell activation^23^, sufficient to stimulate CD56 upregulation on NK cells and activate their cytotoxic function against bladder cancer cells. Importantly, NK cells required other PBMC for this activation, since BCG treatment of freshly isolated NK cells did not enhance effector function against bladder cancer cells^29^. In parallel, cytokines detected in the urine of BCG-treated bladder cancer patients usually decrease quickly and only certain chemokines remain detectable several days after instillations^30^. These findings suggested that BCG could provide beneficial NK priming that, together with extremely low concentrations of cytokines, might support growth, without exhaustion, of B-pNK for anti-tumour cell therapies, providing the basis for lower toxicity regimes^31^ and increase the persistence of the transferred NK cells after in vivo infusions. Here we demonstrate that BCG-primed anti-tumour NK cells incubated with extremely low doses of IL12, 15 and 21 selectively proliferate and maintain their cytotoxic function. BCG-primed IL-stimulated NK (**BIL-NK**) cells overgrew other lymphocyte types to expand 200-fold, without any cell sorting, and killed a broad range of solid tumour cell lines via NKG2D, even after culture for one month in vitro. Remarkably, an unconventional long-lived NKG2C population was also identified after cytokine addition. However, these cells were different to adaptive-like NK cells, generated in HCMV^+^ individuals. Finally, we show that BCG-priming followed IL12, 15 and 21 stimulation can expand and activate in vitro NK cells from immunosuppressed microenvironments, such as bone marrow from paediatric cancer patients. These data identify a potent anti-tumour NK cell population and a novel regime for maintenance of NK cells with enhanced fitness which could be optimised for development of novel cancer cell therapy agents.

## Results

### BCG-primed CD56^high^CD16^+^ NK cells degranulate against multiple types of solid tumours

**Figure 1:**
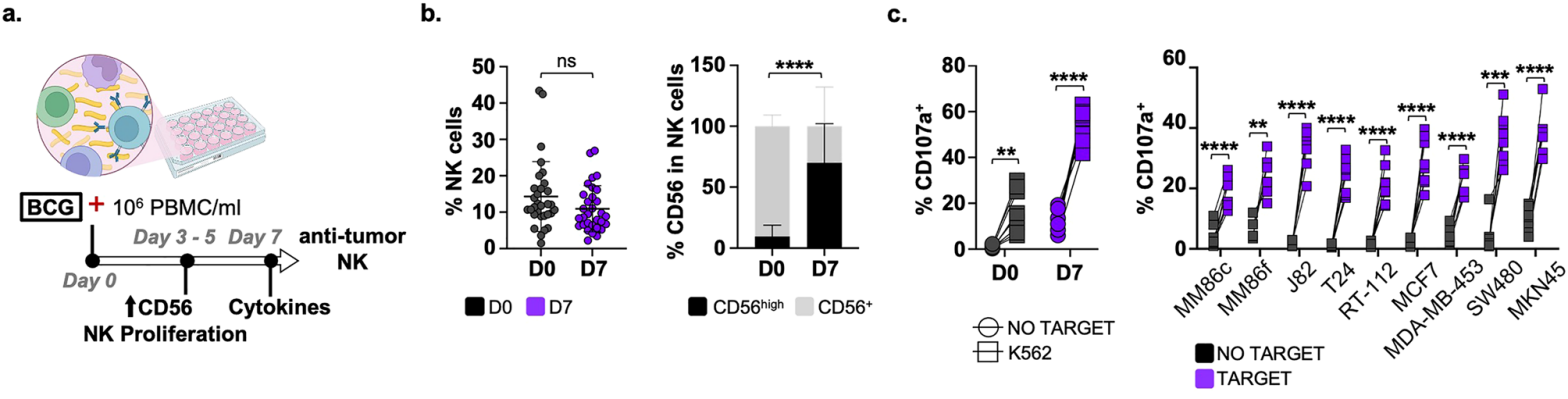
CD56^high^ NK cells kill a wide variety of solid tumour cells after one week in culture. (**a**). Experimental design based on a model for bladder cancer. 3–5 days after co-culture of PBMC with BCG, without addition of exogenous cytokines, NK cells start to proliferate and upregulate CD56. At day 7, several cytokines are detected in the culture and NK cells exhibit potent degranulation against bladder cancer cells. Figure created with Biorender. (**b**). NK cell activation after BCG-priming. PBMC from 31 healthy donors in 15 independent experiments were co-cultured with BCG for a week. Cells were then analysed by flow cytometry to determine the percentage of CD56^high^ NK cells (black) (mean ± SD: 69.9 ± 32%). Statistical analysis was done using paired sample t-test (****p < 0.0001). (**c**). Degranulation of B-pNK cells against different solid tumour cell lines. BCG-activated PBMC were used as effector cells (1:2 E:T ratio, 25,000 NK cells to 50,000 target cells) in degranulation experiments against K562 cells (positive control, left) or solid tumour target cell lines (right): melanoma (MM86c, MM86f.), bladder (T24, J82, RT-112); breast (MCF7, MDA-MB-453); colon (SW480); and gastric (MKN45) cancers. Surface LAMP-1 (CD107a) was measured by flow cytometry. Statistical analysis (n = 8) was done by paired sample t-test **p < 0.01, *p* < 0.05; ***p < 0.001, ****p < 0.0001). (**a**) Experimental design. (**b**) CD56 upregulation. (**c**) Degranulation on D7.

PBMC from healthy donors were used to test whether BCG-stimulated NK cells (B-pNK) could kill a range of solid tumours besides bladder cancer. As previously observed, although the percentage of total NK cells did not increase during this week in the BCG-stimulated PBMC co-culture, the proportion of CD56^high^ cells increased significantly compared to untreated cells (Fig. 1a,b; Fig. S1a). Since these B-pNK cells do not correspond to peripheral blood immature CD56^bright^ NK cells, but rather correspond to activated NK cells, we refer to them as CD56^high^. Degranulation assays showed that BCG-activated NK cells were able to efficiently recognize a panel of different types of solid tumour cell lines (Fig. 1c), suggesting that BCG priming could potentiate NK cell activity against multiple solid tumours, not just bladder cancer.

### BCG-primed NK cells activate the Reactome signalling cascades described for IL12, 15 and 21, but not for IL18

**Figure 2:**
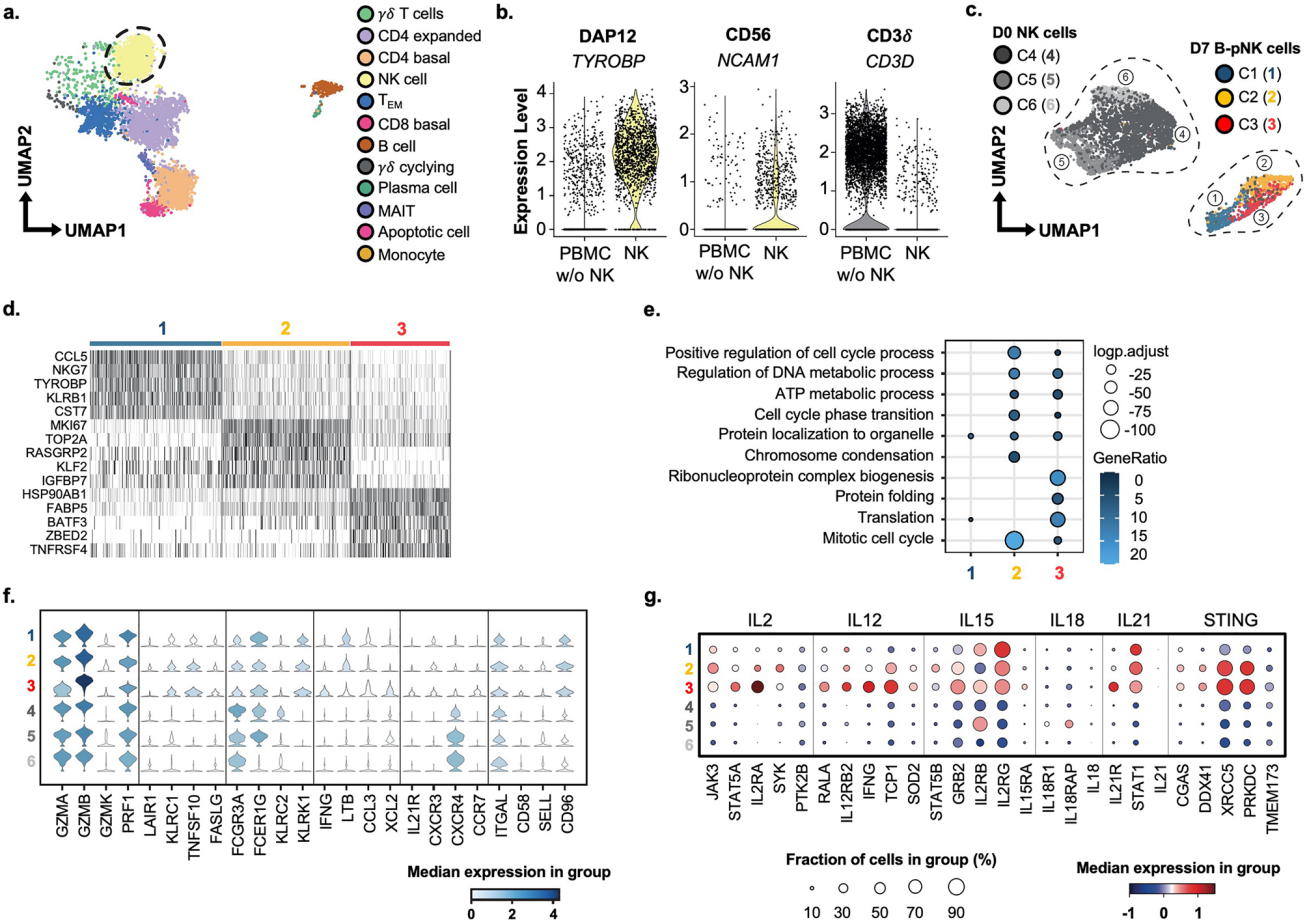
scRNA-seq analysis of B-pNK cells. (**a**). PBMC clusters. UMAP plots represent the 12 clusters identified in BCG-activated PBMC from 3 healthy donors and analysed by scRNA-seq. A dashed circle highlights the NK cell cluster. (**b**). NK cluster. Violin plots confirm the expression of the NK-associated genes, *TYROBP* (DAP12) and *NCAM1* (CD56) within the NK cluster, and *CD3D* (CD3δ) in the PBMC cluster (excluding NK cells) compared to NK cells. (**c**). Differential transcriptome compared to peripheral blood NK cells. UMAP represents three subclusters identified within BCG- primed NK cells (C1, C2, C3) and three subclusters identified within peripheral blood NK cells from 4 healthy donors (C4, C5, C6)^33^. Subcluster annotation was performed using FindClusters and FindMarkers functions from Seurat R package (see scRNA-seq in Materials and Methods). (**d**). Heat-map. Heat-map represents the scaled expression of the 5 most differentially expressed genes in the three NK cell subclusters (C1, C2, C3). (**e**). Selected GO terms. Comparison among the NK cell subclusters (C1, C2, C3) using markers differentially expressed within each cluster. (**f**). Canonical NK cell markers in activated vs resting NK cells. Violin plots analysing the expression of NK-related molecules in BCG-primed NK cell subclusters (C1, C2, C3) compared to peripheral blood NK cell subclusters (C4, C5, C6). (**g**). Reactome enriched pathways. Dot plots illustrate the expression of some of the most important genes annotated within each interleukin pathway (IL2, 12, 15, 18, and 21) and cGAS-STING signalling pathway among all NK cell subclusters (C1-6).

To further explore the mechanism of action of BCG in PBMC cultures, in-depth characterization of BCG-primed cultures from three healthy donors were performed by scRNA-seq analysis^32^. 12 clusters were defined, based on differential gene expression, showing several activated populations, including NK cells, CD4 T, T_EM_ and MAIT cells (Fig. 2a, Fig. S1b). The markers used to define the NK cell subpopulation in this scRNA-seq analysis are shown in Fig. 2b. To better define the effect of BCG priming on NK cells, these data were compared with publicly available scRNA-seq data from resting peripheral blood NK cells^33^. Quality control and cell numbers of both BCG-activated and resting NK cells are shown in Fig. S2a,b, respectively. After clustering, the merged Uniform Manifold Approximation and Projection (UMAP) plots clearly showed that BCG-stimulated (C1-3) and resting (C4-6) NK cells were located separately within the 2D projection, due to their different transcriptomic signatures (Fig. 2c, Fig. S2c). Within B-pNK cells, cluster 1 (C1) had features consistent with a non-proliferating CD56^dim^ subset while clusters C2 and C3 corresponded to highly proliferative and activated cells (Fig. 2d,e). Interestingly, BCG-priming also upregulated pathways related to cell cycle and ATP-metabolism (Fig. 2e). Stacked violin plots were built to compare differential expression of key NK molecules (Fig. 2f). Cells in C3 differentially expressed genes associated with strong cytotoxic capacity, such as *GZMB* and *XCL*2. Transcripts for CD94 (*KLRC1*) and CD16 (*FCGRIIIA*) proteins identified in B-pNK by flow cytometry were also present, although the relative abundance of RNA and protein differed to some extent.

All three clusters, C1-3, showed enriched expression of cytotoxicity-related transcripts, such as granzymes, perforins, TRAIL (*TNFSF10)*, *FASLG, IFNG, LTB*, *CCL3* and NKG2D (*KLRK1*). However, B-pNK cells did not have a clearly defined memory-like phenotype, since NKG2C (*KLRC2*) was not expressed and *FCER1G* was found at levels comparable to resting cells. Another transcript clearly upregulated in C1-3 clusters was the chemokine receptor CXCR3, that could facilitate migration to the tumour environment mediated by CXCL9 and 10.

Since we aimed to investigate the possibility of further expanding in vitro these anti-tumour NK cells, the gene expression signatures of interleukin pathways described in the Reactome pathway database were analysed in all NK clusters (Fig. 2g, Fig. S2d,e). Surprisingly, neither the IL18 receptor, nor its signalling cascade, were upregulated after BCG-priming. In contrast, expression of molecules involved in the activation of receptors for IL12, 15 and 21 was clearly increased after BCG treatment. These data suggest that B-pNK cells could respond to this combination of cytokines.

Overall, the data from these analyses reveal that B-pNK cells show a particular activation phenotype together with capacity to respond to cytokines and to migrate, features well suited for anti-tumour activity.

### Minimal concentrations of IL12, 15 and 21 mediate selective proliferation of BCG-primed NK cells

To evaluate the capacity of NK cells to proliferate in the cytokine combination described above, we firstly established minimal concentrations supporting NK cell growth, but avoiding overstimulation and potential cell exhaustion. Titration experiments were performed incubating PBMC for 7 days with just one dose of individual or combined IL12, 15, 18 and 21 cytokines on day 0. A minimal dose of IL21, together with IL15 and IL12 [0.1 ng/ml IL12, 0.5 ng/ml IL15, and 0.5 ng/ml IL21], yielded the highest number and proportion of anti-tumour CD56^high^CD16^+^ NK cells, after 7 days in culture (Fig. S3).

**Figure 3:**
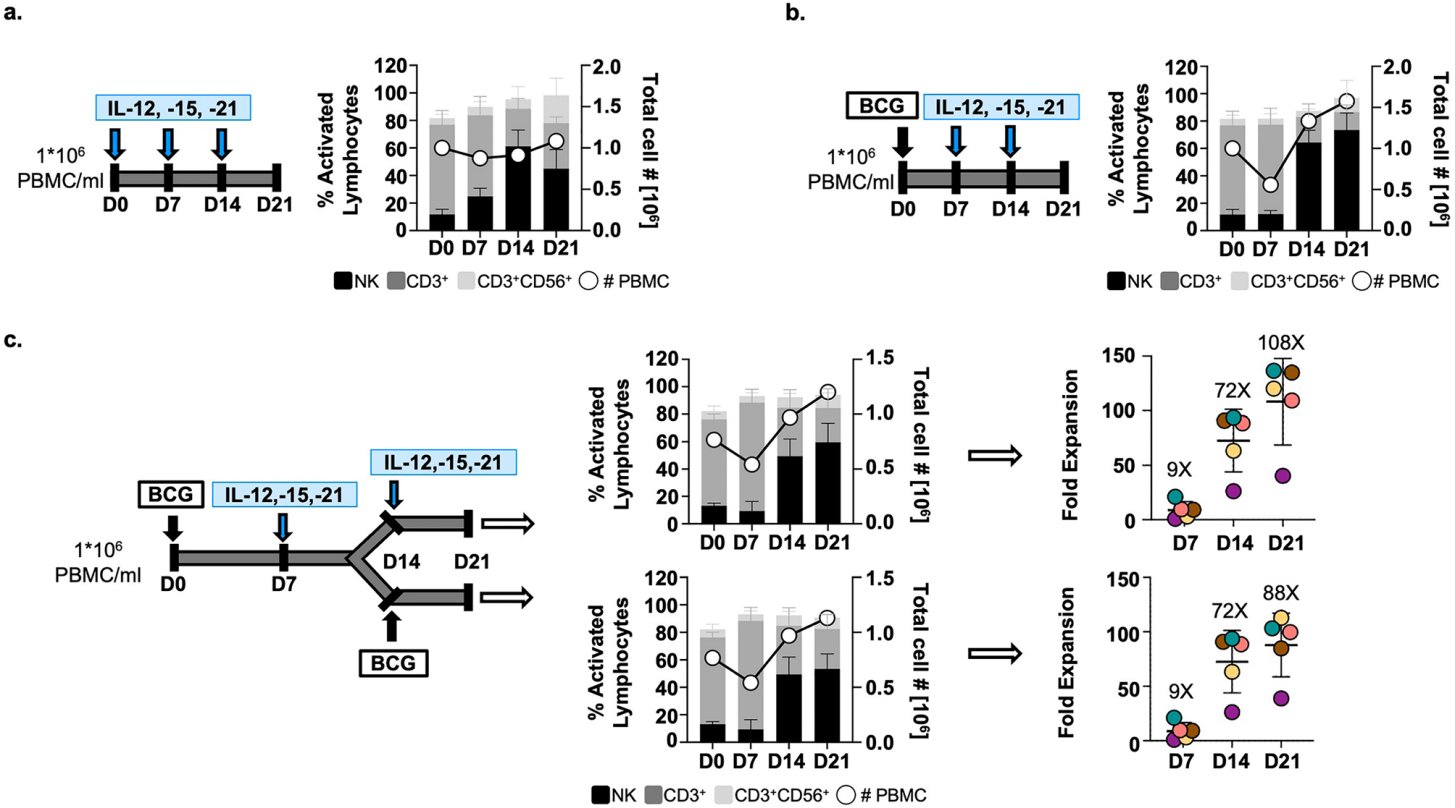
BCG priming enhances selective proliferation of NK cells. a, b. BCG vs low-dose cytokine priming. PBMC from 4 healthy donors were incubated for a week either with minimal-dose IL12, 15, and 21 (**a**) or with BCG and then weekly stimulated with minimal-dose cytokines (**b**); experimental designs are depicted for each case. Cells were counted (◯, right Y axis) and analysed by flow cytometry weekly. The mean percentages and SD of activated lymphocytes (left Y axis) are shown, as well as the different lymphocyte subsets, depicted with different shades of grey, as indicated. (**c**). BCG vs low-dose cytokine re-stimulation. PBMC from 5 healthy donors were incubated with BCG and stimulated with either minimal-dose IL12, 15, and 21 or with BCG on day 14. Cells were counted (◯, right Y axis), the different lymphocyte subpopulations were determined by flow cytometry (left Y axis), and the CD56^high^ NK cells fold expansion was calculated considering % NK cells and the number of live cells in the culture; this number was compared to the initial % CD56^bright^ NK cells. Each donor is represented in a different colour in a scatter plot (mean ± SD error bars). (**a**) Low-dose cytokines. (**b**) BCG + low-dose cytokines. (**c**) NK cell expansion ±BCG for 21 days.

Long-term PBMC cultures were then set up to test the possibility of expanding anti-tumour NK cells with low doses of IL12, 15, 21 and whether BCG provided any advantage (Fig. 3a,b). Indeed, a higher proliferation (right axes) and, importantly, a stronger increase in NK cell percentage (left axes) was obtained when cultures were primed with BCG. The robust expansion of NK cells from BCG-primed IL-stimulated NK cells (BIL-NK) was consistent in PBMC cultures (n = 20) (Fig. S4a) and was also higher than that of cultures stimulated with higher doses of IL12, 15, and 18, refreshing regularly the culture with IL15 as described by^20^ (Fig. S4b–e). Interestingly, re-challenging the cultures with BCG, one week after cytokine stimulation (day 14) showed no increase in NK cell numbers nor stimulation phenotype, ruling out a memory feature in this system (Fig. 3c).

These results demonstrate that BCG-priming confers a specific proliferative capacity to NK cells, overgrowing other PBMC when cultured with low concentration of IL12, 15, and 21.

### BCG-primed NK cells, expanded by weekly addition of minimal cytokine doses, are cytotoxic against solid tumour cells after one month in culture

**Figure 4:**
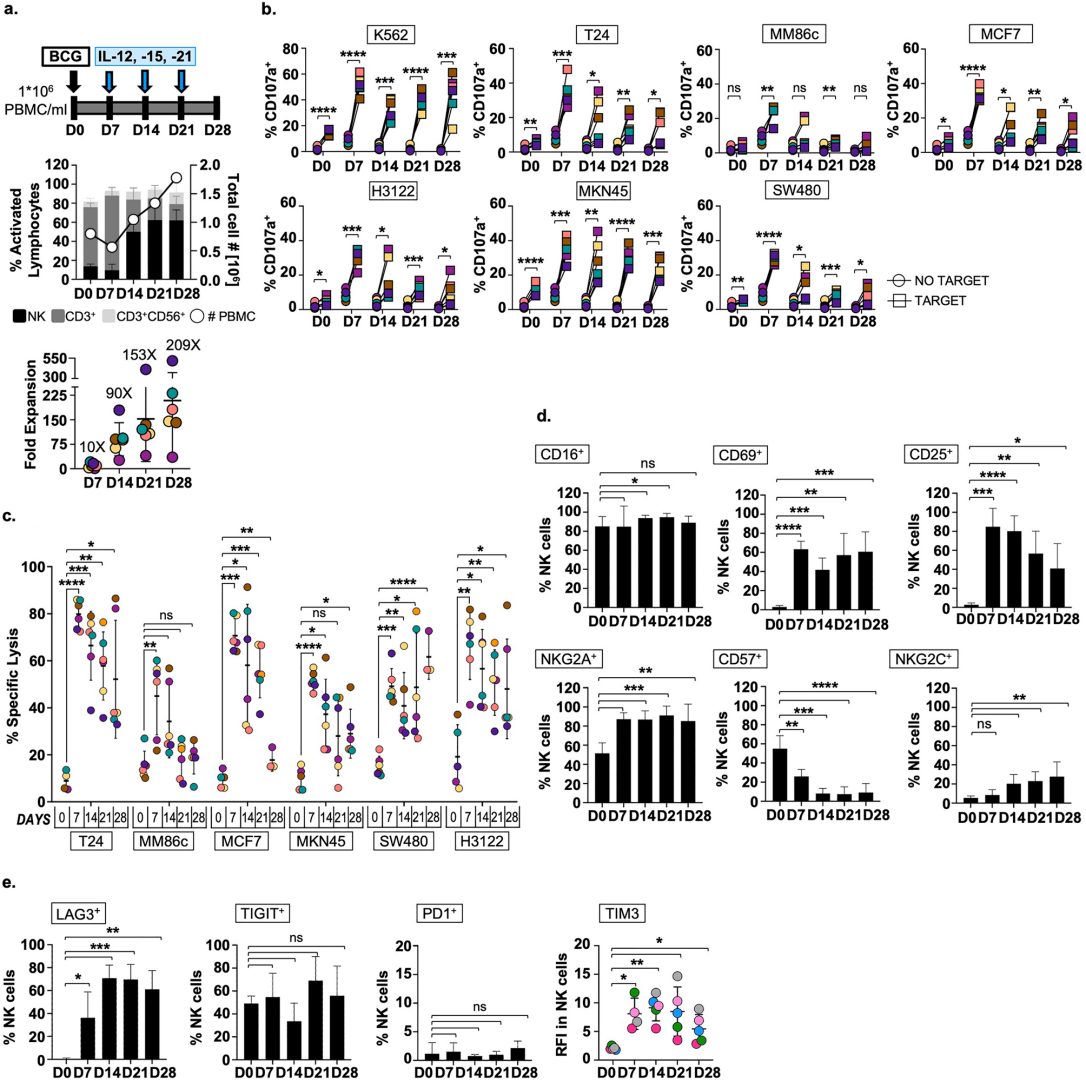
BIL-NK cells proliferate for at least one month and maintain effector functions. (**a**). Expansion after four weeks in vitro. PBMC from 6 healthy donors were incubated with BCG and stimulated with weekly minimal-dose IL12, 15, and 21 for 28 days. Cells were counted ◯, right Y axis) and the different subsets analysed by flow cytometry (left Y axis). BIL-NK expansion was calculated as explained for Fig. 3c. Fold expansion value for each donor (different colours) is represented in a scatter plot (mean ± SD error bars). (**b**), (**c**). Degranulation and cytotoxicity assays. NK cells were tested as effector cells against solid tumour target cell lines bladder (T24) melanoma (MM86c), breast (MCF7), gastric (MKN45), colon (SW480), and lung (H3122) cancers. K562 were used as positive control. For degranulation (**b**), a 1:2 E:T ratio (NK to target) was used, and surface LAMP-1 (CD107a) was measured by flow cytometry. For cytotoxicity assays (**c**), effector NK cells were incubated with solid tumour target cells labelled with calcein-AM (5:1 E:T ratio). % of specific lysis for each donor (different colours) is represented in a scatter plot (mean ± SD error bars). Statistical analyses were done by paired sample t-tests (*p < 0.05, **p < 0.01, ***p < 0.001, ****p < 0.0001). (**d**), (**e**). Activation, maturation, and immune checkpoint profile. NK cells were recovered weekly and analysed for the indicated panel of receptors associated to activation and maturation (**d**) and exhaustion (**e**) by flow cytometry. Bar graphs and scatter dot plot show average and SD error bars of the % of NK cells expressing each marker are depicted for 6 healthy donors (**e**) and 5 healthy donors (**e**) from two different experiments. For TIM3 (**e**), because the whole NK population had a single peak, the different levels of expression are shown as relative fluorescence intensity (RFI): RFI = MFI sample/MFI IgG isotype control (negative control), where MFI is mean fluorescence intensity. Statistical analysis of NK cells expressing each marker against basal expression (D0) was done by paired t-test (*p < 0.05, **p < 0.01, *p* < 0.05; ***p < 0.001, ****p < 0.0001). (**a**) Expansion after 28 days. (**b**) Degranulation assays. (**c**) Cytotoxicity assays. (**d**) Phenotype after 28 days. (**e**) Immune checkpoint expression after 28 days.

B-pNK cells grown long-term with minimal-dose cytokines, BIL-NK, were characterised for phenotype and function. BCG-primed PBMC from 6 healthy donors were cultured for one month, with just weekly cytokine addition. Cell numbers were still expanding at day 28 and NK cells became, on average, 62% of the culture (Fig. 4a); a 209-fold expansion (35- to 517-fold) from their initial number in PBMC. Each week, 7 days after stimulus, an aliquot of cells was recovered and tested in degranulation and cytotoxicity assays against bladder, melanoma, breast, gastric, colon and lung cancer cell lines (Fig. 4b,c). BIL-NK cells kept their anti-tumour capacity over this prolonged in vitro culture. Importantly, functional assays were done 7 days after refreshing the culture with cytokines and no other survival or function enhancer was added since, suggesting a prolonged stimulation over time.

The phenotype of these long-lived effector NK cells was characterized by flow cytometry (Fig. 4d). As previously described, after one week with BCG^23^, at day 28 CD56^high^ NK cells expressed CD16^+^ and NKG2A^+^. Activation was confirmed by expression of CD25 and CD69 in a high percentage of NK cells, although CD25 started to decrease at 21 days. Surprisingly, after cytokine stimulation, around 22% of activated NK cells became positive for NKG2C at day 14, and this percentage grew even higher the following weeks, although this marker was absent on day 7 after BCG priming. Moreover, this phenotype was not accompanied by an increase in CD57 expression. Finally, a selection of receptors associated with inhibitory capacity was evaluated (Fig. 4e, Fig. S5). Remarkably, despite expression of LAG3 or TIM3, NK cells did not show any signs of senescence, as they continued to proliferate and efficiently mediate cytotoxic function for the whole month.

Taken together, these results confirm that exposure of PBMC to BCG followed by weekly stimulations with minimal-dose IL12, 15, and 21 cytokines dramatically expands a cytotoxic, long-lived CD56^high^CD16^+^NKG2A^+^ NK cell subset which remains fit for target recognition and killing after 28 days in culture without becoming exhausted.

### BCG-primed NK cells eliminate tumours through NKG2D activation and can secrete cytokines

**Figure 5:**
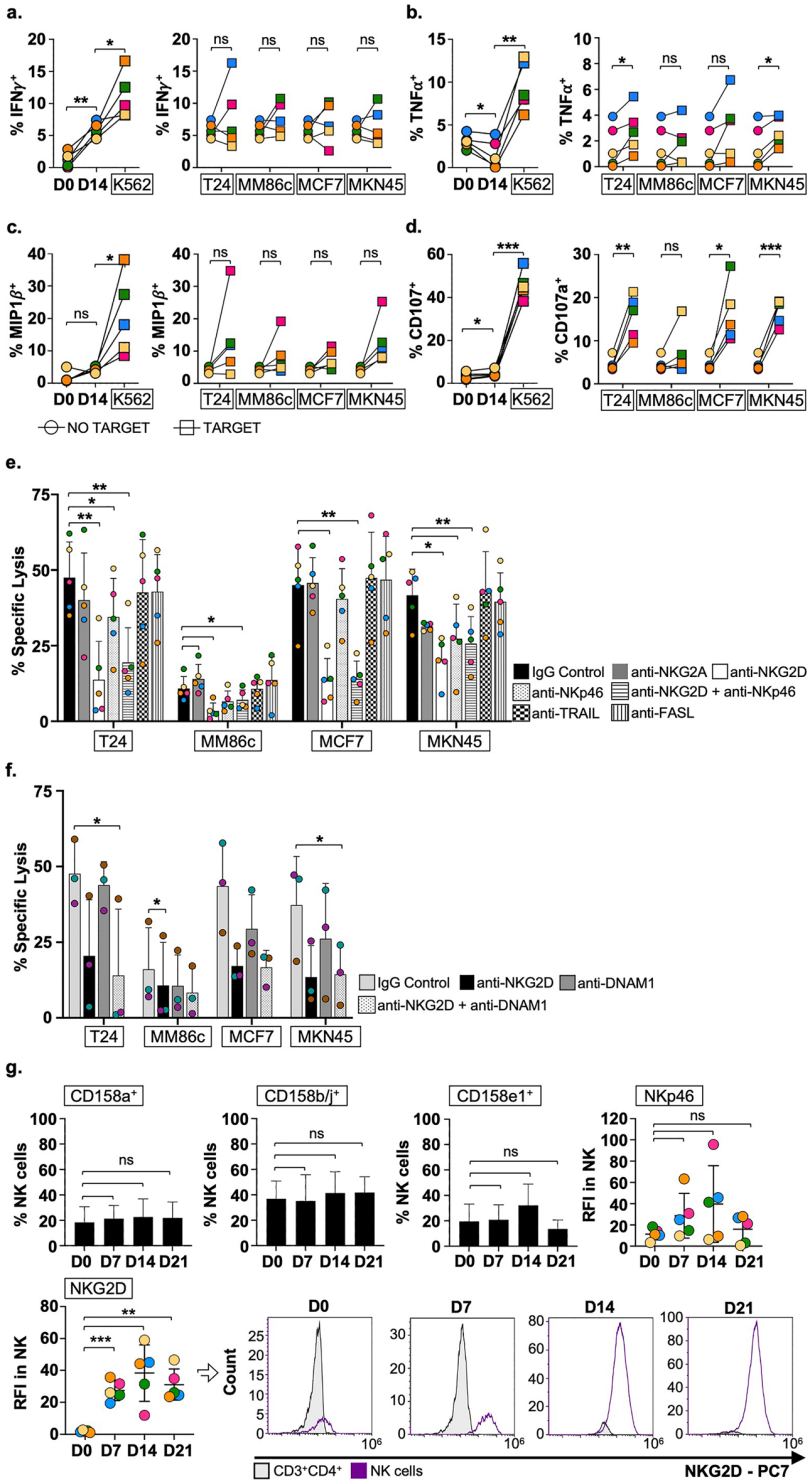
Anti-tumour cytotoxicity of effector BIL-NK cells is NKG2D-mediated. PBMC from 5 healthy donors (different colours) were primed with BCG, stimulated weekly with minimal-dose IL12, 15, and 21 and used in functional assays at day 14 (2 different experiments). (**a**–**d**). Intracellular cytokine-release assays and degranulation. BIL-NK cells were tested as effector cells (1:2 E:T ratio, NK to target) against solid tumour target cell lines bladder (T24) melanoma (MM86c), breast (MCF7), and gastric (MKN45) cancer without MHC-I blocking. Response against K562 cell line on day 14 was used as positive control. Intracellular IFNγ (**a**), TNFα (**b**), and MIP-1β (**c**) and surface LAMP-1 (CD107a) (**d**) were measured by flow cytometry. Statistical analysis compared to basal expression (D0) and no target on D14 was done by paired t-test (*p < 0.05, **p < 0.01, p < 0.05; ***p < 0.001, ****p < 0.0001). (**e**), (**f**). Cytotoxicity assays with blocking antibodies. Effector PBMC were treated with anti-NKG2A, NKG2D, NKp46, TRAIL and FASL blocking antibodies before incubation with solid tumour target cells labelled with calcein-AM (10:1 E:T ratio) (**e**). In a separate experiment, BCG-primed NK cells from 3 healthy donors were treated with anti-DNAM1 and anti-NKG2D blocking antibodies and tested against solid tumour targets (**f**). No anti-MHC-I antibody was used in either experiment. Anti-IgG treatment was used as control of cytotoxic activity. (**g**). Phenotype characterization after three weeks in culture. BIL-NK cells were analysed for the indicated panel of receptors at day 21 by flow cytometry. Average and SD error bars of the percentage of NK cells expressing each marker are depicted as bar graphs or scatter dot plots. For NKG2D and NKp46, because the whole NK population had a single peak, the different levels of expression are shown as relative fluorescence intensity (RFI): RFI = MFI sample/MFI CD3^+^CD4^+^ (negative control), where MFI is mean fluorescence intensity. Representative histograms for the analysis of NKG2D expression are shown. Statistical analysis of NK cells expressing each marker against basal expression (D0) was done by paired t-test (*p < 0.05, **p < 0.01, *p* < 0.05; ***p < 0.001, ****p < 0.0001). (**a**) IFNγ-release on D14. (**b**) TNFα-release on D14. (**c**) MIP-1β-release on D14. (**d**) Degranulation assays on D14. (**e**) Activating receptor blockade(cytoxicity assay; D14). (**f**) DNAM-1 blockade(cytoxicity assay; D14). (**g**) NK receptors on NK cells.

Experiments to further understand the functional capacities of BIL-NK were performed next. BCG-primed PBMC from 5 healthy donors were cultured for three weeks with cytokine stimulations. Functional assays were carried out on day 14. Without a target, BIL-NK increased slightly the release of IFNγ compared to day 0, even without any re-stimulation prior to experiments. However, production of IFNγ, TNFα and MIP-1β (Fig. 5a,b,c) was not significantly increased by target recognition. In contrast, BIL-NK cells efficiently degranulated against bladder, breast, and gastric tumour cell lines (Fig. 5d). To investigate the receptors involved in BIL-NK mediated tumour killing, cytotoxicity assays using blocking antibodies for NKG2D, NKG2A, NKp46, TRAIL and FASL receptors were performed next (Fig. 5e). A significant decrease of specific lysis occurred when NKG2D was blocked either alone or in combination. NKp46 blockade only significantly decreased the killing of bladder and gastric cancers, however the effects of NKG2D and NKp46 were not additive. Interestingly, blocking NKG2A, TRAIL or FASL did not affect the killing capacity of the effector cells. Finally, the role of DNAM1 was assessed (Fig. 5f). Although DNAM1 blockade also reduced BIL-NK response to all the cancer cell lines tested, these changes in specific lysis did not reach statistical significance. In agreement with these functional data, NKG2D was strongly upregulated after BCG-priming and this high expression was maintained after cytokine expansion, while KIRs and NKp46 expression was stable (Fig. 5g).

All together these data suggest that BCG priming followed by minimal dose cytokine stimulation expands a population of activated NK cells with strong anti-tumour cytotoxic capacity mediated primarily by an upregulated expression of NKG2D and, to a lesser extent by DNAM1 and NKp46, and independent of the expression of the inhibitory receptors^24^.

### NKG2C expression on BIL-NK cells is independent of cytomegalovirus seropositivity

The significant increase in NK cell surface NKG2C^+^ expression, occurring after cytokine incubation of B-pNK, was surprising. So, the expression of NKG2C was further analysed.

**Figure 6:**
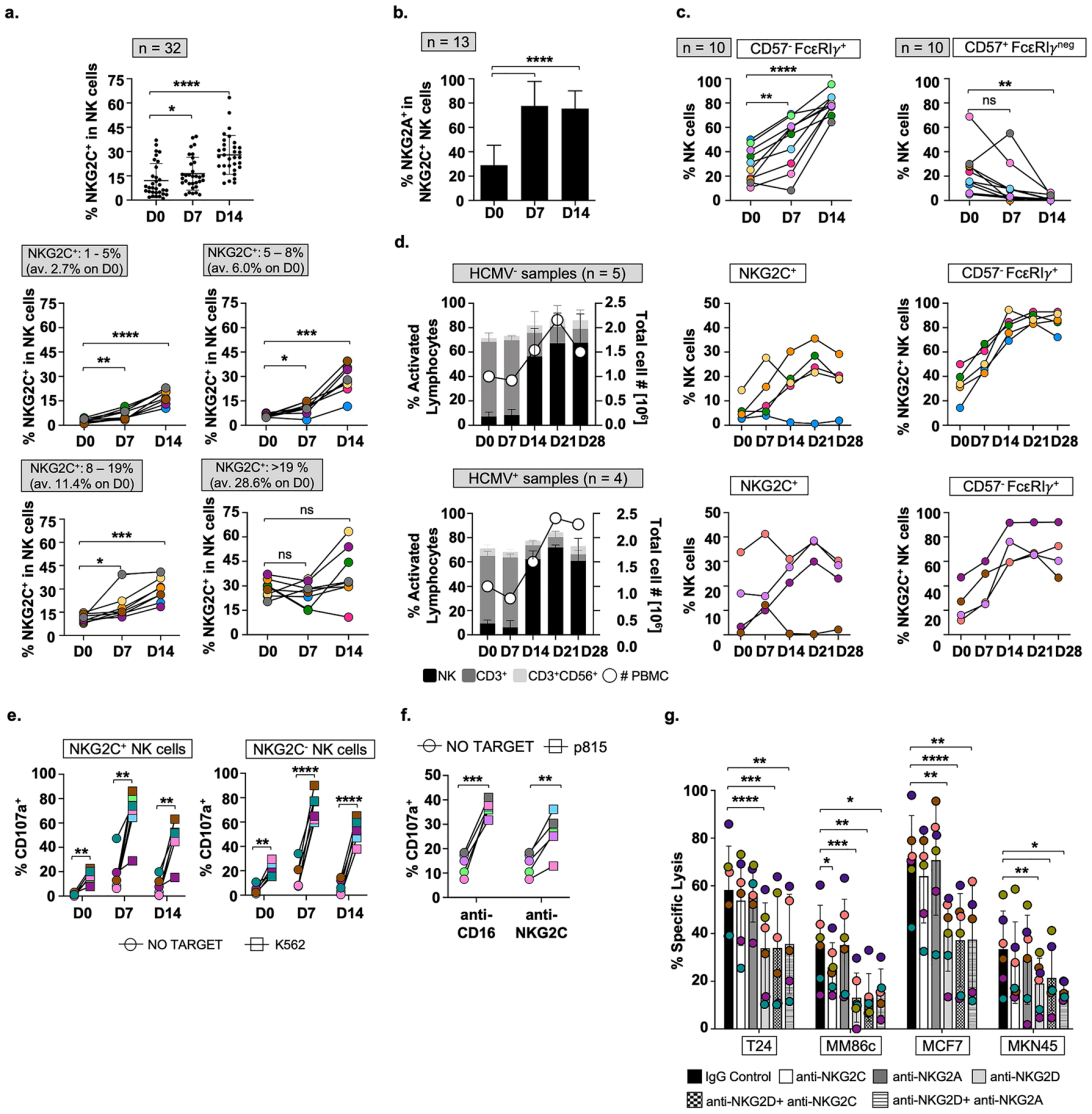
BIL-NK cells upregulate NKG2C expression regardless of HCMV-serology. (**a**). NKG2C expression after two weeks in vitro. PBMC were incubated with BCG for one week and then stimulated with minimal-dose IL12, 15, and 21. Results were obtained from 8 independent experiments involving 32 healthy donors divided in 4 groups (n = 8 per group; different colours) according to basal NKG2C expression (D0) on NK cells. (**b**), (**c**). NKG2C^+^ BIL-NK cell subsets. Bar graph shows mean % and SD error bars of NKG2A^+^ within the NKG2C^+^ subset (n = 13) (**b**). Scatter plots represent % of CD57^-^FcεRIγ^+^ and CD57^+^FcεRIγ^-^ subsets within the NKG2C^+^ region (n = 10) (**c**). Statistical analysis against D0 was done by paired t-test (*p < 0.05, **p < 0.01, *p* < 0.05; ***p < 0.001, ****p < 0.0001). (**d**). BIL-NK cells from HCMV–known serology donors. PBMC from 5 HCMV^-^ and 4 HCMV^+^ donors (each in a different colour) were incubated with BCG and stimulated with weekly minimal-dose IL12, 15, and 21 for 4 weeks. Cells were counted and the subsets analysed as explained previously. Scatter plots represent upregulation of NKG2C expression within the NK region and % of CD57^-^FcεRIγ^+^ cells within the NKG2C^+^ NK cell region. (**e**), (**f**). Degranulation assays. NK cells (1:2 E:T ratio) from 6 healthy donors (different colours) were tested against K562. Surface LAMP-1 (CD107a) was measured by flow cytometry within the NKG2C^+/-^ NK cells (**e**). For redirected degranulation, 25000 effector BIL-NK cells (1:2 E:T ratio) from 5 healthy donors were tested against target p815 mouse mastocytoma cells treated with either NKG2C antibody or CD16 as positive control (**f**). Statistical analysis (2 independent experiments) comparing degranulation against no target was done by paired t-test (*p < 0.05, **p < 0.01, *p* < 0.05; ***p < 0.001, ****p < 0.0001). (**g**). Cytotoxicity assays with blocking antibodies. Effector PBMC from 6 healthy donors (different colours) were treated with anti-NKG2C, NKG2A, and NKG2D antibodies before incubation with target cells labelled with calcein-AM (10:1 E:T ratio) without anti-MHC-I blockade (2 independent experiments). Statistical analysis comparing against anti-IgG treatment (cytotoxic activity control) was done by paired t-test (*p < 0.05, **p < 0.01, *p* < 0.05; ***p < 0.001, ****p < 0.0001). (**a**) Expansion of NKG2C^+^. (**b**)NKG2A^+^ expression. (**c**) CD57 and FcεRIγ in NKG2C^+^. (**d**) Expansion in HCMV^+/-^ samples after 28 days. (**e**) Degranulation vs. K562. (**f**) Redirected ADCC on D14. (**g**) Receptor blockade(cytotoxicity assay; D14).

32 healthy donors with different initial frequencies of NKG2C^+^ NK cells were incubated for a week with BCG, stimulated with minimal-dose IL12, 15, and 21 cytokines for a second week and the expression of NKG2C was determined in the NK cell population at day 14 (Fig. 6a; Fig. S6a, b). The percentage of NKG2C^+^ NK cells increased in all the donors tested. This expansion did not occur the first week after BCG priming, but rather during the subsequent week of stimulation with low doses of cytokines. Analysis was performed according to the NKG2C basal expression of each donor at day 0. Interestingly, low and medium NKG2C basal expression donors (NKG2C^+^: 1–19% D0) more than tripled their NKG2C^+^ NK cell population on day 14. However, those donors with a high NKG2C^+^ percentage in resting cells (NKG2C^+^: > 19% D0), generally maintained the % of this population. As most B-pNK also express NKG2A, most BIL-NK cells are double positive for NKG2C^+^ and NKG2A^+^ (Fig. 6b), in contrast to previously described human cytomegalovirus (HCMV)-associated NKG2C^+^ NK cells which do not express the inhibitory CD94 counterpart^34^. In fact, in donors with high basal expression of NKG2C, this population was mainly NKG2A negative at day 0, suggesting that the population generated after BCG-priming and cytokine stimulation is a novel subset of NK cells. Representative NKG2C^+^ NKG2A^+^ flow cytometry plots from 3 donors with either low, medium or high initial frequencies of NKG2C^+^ NK cells are shown in Fig. S6c. Moreover, when effector cells from 10 donors with different initial percentages of NKG2C^+^ NK cells were analysed, most of the expanded NKG2C^+^ NK cells were CD57^-^ and FcεRIγ^+^, in contrast to the previously described adaptive-like CD57^+^ and FcεRIγ^neg^ phenotype, which decreased over time in these cultures (Fig. 6c; Fig. S6d). Because BCG followed by low dose cytokine stimulation results in strong proliferation of NK cells, our data showing expression of FcεRIγ at day 14 confirm the presence of this protein in proliferating cells^35^.

Since it appears that a novel NKG2C^+^ population is expanded upon cytokine stimulation after BCG-priming, expression of this receptor was evaluated in 9 donors with known serology for HCMV (Fig. 6d). NK cells from both HCMV^+^ and HCMV^-^ donors increased the expression of NKG2C, as well as a CD57^-^ and FcεRIγ^+^ population, suggesting that the expansion of this subset is independent of HCMV status.

To test whether CD94/NKG2C could play an activating role in BIL-NK cells, functional assays were performed. Degranulation experiments demonstrate that both NKG2C^+^ and NKG2C^-^ cells are active upon target recognition (Fig. 6e) and redirected antibody directed cell cytotoxicity (ADCC) assays show that NKG2C receptor ligation activates killing (Fig. 6f). Specific lysis assays were done in the presence and absence of blocking antibodies recognising NKG2A, NKG2C and NKG2D (Fig. 6g). 6 donors with different frequencies of NKG2C-expressing NK cells at day 0, were incubated for a week with BCG, stimulated with minimal-dose cytokines for a second week and the expression of NKG2C was determined in the NK cell population at day 14 (Fig. S6e, table). Again, a significant decrease of cytotoxic activity against a variety of solid tumours occurred when blocking NKG2D receptor. Interestingly, NKG2A blocking did not increase killing capacity, ruling out NKG2A inhibition in this system.

### NK cells from cancer patients proliferate and recover their effector function after BCG-priming and minimal-dose cytokine stimulation

**Figure 7:**
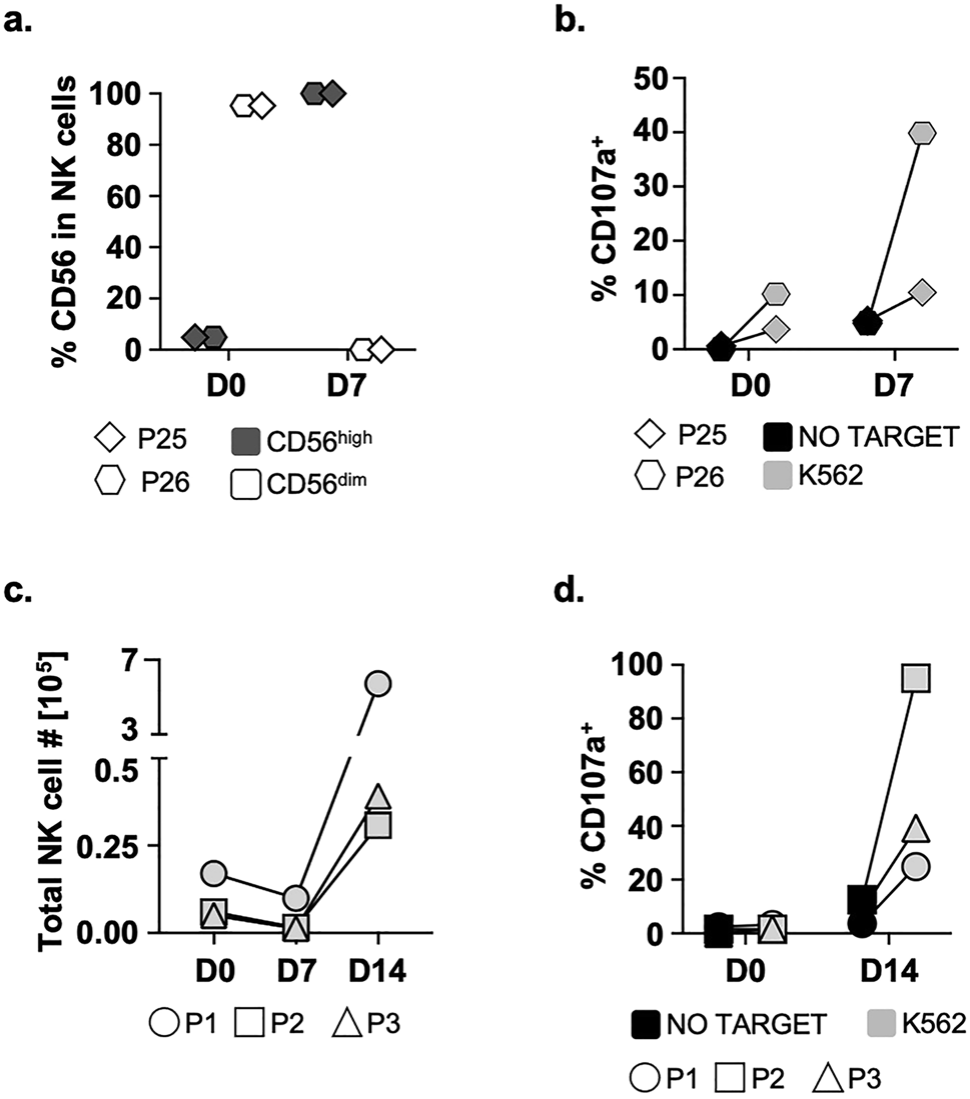
BCG-priming rescues effector functions of NK cells from cancer patients. (**a**), (**b**). NMIBC patients. PBMC from 2 bladder cancer patients receiving intravesical instillations of BCG (T1G3) were co-cultured with BCG in vitro. Cells were analysed by flow cytometry before (D0) and after 7 days in culture with BCG (D7). Upregulation of CD56 expression in total NK cells was plotted as a measure of activation (**a**). For degranulation (**b**), PBMC were used as effector cells (5:1 E:T ratio, 25,000 PBMC to 50,000 target cells) against K562 on day 0 and day 7. Surface LAMP-1 (CD107a) was measured by flow cytometry. Each donor is represented with a different symbol. (**c**), (**d**). Cancer paediatric patients. BMMC cells from a CNS myeloid sarcoma cancer patient (P1) and 2 B-ALL patients (P2 and P3) were co-cultured with BCG for one week and then stimulated with minimal-dose IL12, 15, and 21. For NK cell expansion (**c**), cells were recovered, counted, and analysed by flow cytometry on days 0, 7 and 14. Total NK cell number was calculated considering the percentage of NK cells and the number of live cells in the culture. For degranulation (**d**), NK cells were used as effector (1:2 E:T ratio, 12000 NK cells to 24000 target cells) against K562 on day 0 and day 14. Surface LAMP-1 (CD107a) was measured by flow cytometry. Each donor is represented with a different symbol. (**a**)NMIBC: CD56 upregulation. (**b**)NMIBC: Degranulation vs.K562. (**c**)NK expansion. (**d**) Degranulation vs.K562.

BCG-priming followed by minimal-dose IL12, 15, and 21 maintenance can confer potent anti-tumour properties to NK cells from healthy PBMC without reaching exhaustion. So, we explored whether this strategy could recover the cytotoxic capacity of dysfunctional NK cells from cancer patients. Firstly, PBMC samples from two adult NMIBC patients were incubated with BCG for a week and analysed by flow cytometry. NK cell activation was evidenced by upregulation of CD56 expression (Fig. 7a) and enhanced degranulation capacity (Fig. 7b). Next, unfractionated bone marrow mononuclear cells (BMMC) samples from cancer paediatric patients were co-cultured with BCG and stimulated after 7 days in culture with minimal doses of IL12, 15, and 21. Two weeks later, NK cells from B-ALL BM samples expanded from 0.6% on D0 to 7–11% on D14, and NK cells from a patient with CNS myeloid sarcoma expanded from 1.7 on D0 to 44% on D14 (Fig. 7c, Fig S7). These NK cells degranulated on exposure to K562 lymphoblast cells (Fig. 7d). These data show that cytotoxic BIL-NK can be generated from PBMC from an immunosuppressed environment, suggesting that BCG-priming with a particular combination of minimal-dose cytokines is a strategy capable of triggering anti-tumour capacities from dysfunctional NK cells.

These data suggest that it is theoretically possible to envision a therapeutic use of BIL-NK cells.

**Figure 8:**
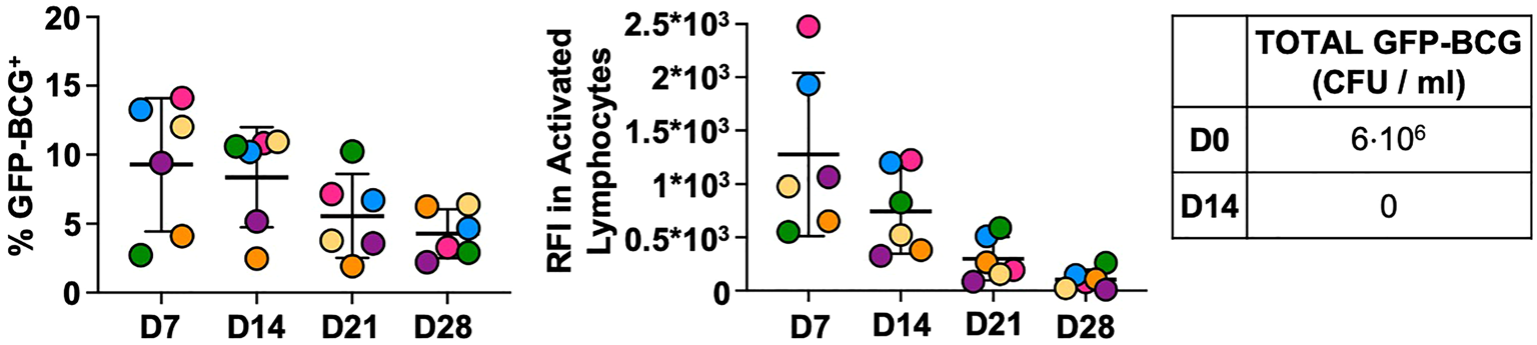
BCG survival in PBMC co-cultures. 10^6^ PBMC from 6 healthy donors (different colours) were co-cultured with 6·10^6^ live GFP-BCG and weekly stimulated with minimal-dose IL12, 15, and 21 cytokines. Cells were recovered at the indicated time points (X axis) and analysed by flow cytometry. The % GFP-BCG present in the activated lymphocyte gate (left) and relative fluorescence intensity [(RFI): RFI = MFI GFP^+^/MFI GFP^-^, where MFI is mean fluorescence intensity] (middle) are plotted for each donor in a scatter plot (mean ± SD error bars). On day 14, cells were recovered and separated by centrifugation at 150 × g. Supernatant was then centrifuged at 850 × g. Supernatant and cells were plated for CFU count. The table (right) shows bacterial GFP-BCG added to the co-culture on day 0 and the colony count (CFU) on day 14 (SN and cells). In these experiments, GFP could be either intracellular or extracellular.

Finally, even though the percentage of live bacteria added to the culture was below 0.1%, the potential administration of whole mycobacteria to cancer patients remained a safety concern. Therefore, the proliferation capacity of BCG in vitro after minimal-dose cytokine stimulation was explored using more stringent conditions, that is, using live bacteria that expressed GFP, for visualization (Fig. 8, Fig. S8). PBMC co-cultured with 6·10^6^ viable GFP-BCG, weekly stimulated with minimal-dose IL12, 15, and 21 were analysed by flow cytometry. On day 14, colony forming units (CFU) were counted and no viable BCG-GFP was recovered from either cell pellets or supernatants (CFU = 0).

Altogether, the progressive decrease in GFP fluorescence and the lack of colony-forming activity confirmed that the BCG gradually died out in these in vitro cultures.

## Discussion

The data presented here demonstrate that culture with mycobacteria followed by minimal doses of a selection of cytokines activates a specific NK subpopulation, which selectively proliferates, and acquires a particular phenotype and a broad anti-tumour capacity, mainly via NKG2D. Further, preliminary data demonstrate the capacity of BCG-priming and minimal-dose cytokine stimulation to rescue dysfunctional NK cells cancer patients.

Pathway analysis of the scRNA-seq data indicates that the mechanisms underlying NK activation after BCG priming, include positive regulation of cell-cycle and metabolic processes as well as enhanced responsiveness to IL12, 15 and 21 stimulations. These observations led to weekly maintenance of BCG-primed PBMC with minimal doses of these cytokines, allowing the growth and enhanced expansion, for at least one month, of functional anti-tumour BIL-NK cells able to recognise several types of solid tumours. Although effector BIL-NK cells share some features with CIML NK cells, the IL18 receptor pathway is not upregulated. Also, BIL-NK cells did not need re-stimulation with cytokines or BCG to better expand or recognise tumours. An NKG2C^+^ CD57^-^ FcεRIγ^+^ subset also expanded in these cultures, suggesting that they are enriched in an alternative anti-tumour activated NK cell subtype. The novel strategy for the expansion of potent anti-tumour NK cells presented here has potential practical implications, establishing conditions to increase the number and fitness of cytotoxic NK cells, effective against a variety of solid tumours, as desired for adoptive cell therapies.

The use of extremely low concentrations of cytokines to expand B-pNK cells was motivated by the observation that only very low levels of soluble factors were found in urine of BCG-treated bladder cancer patients^30^ or released from BCG-activated PBMC in vitro^23^, together with the possibility that excessive chronic cytokine exposure can, in many instances, result in cell dysfunction^36^. Indeed, our data demonstrate that BCG-primed cytotoxic CD56^high^ CD16^+^ NKG2A^+^ NK cells remain fit for target recognition and killing over extended times in culture without becoming exhausted. This outcome was achieved by decreasing cytokine concentrations to the lowest possible level allowing cell survival, around 100 times lower than earlier protocols of NK cell activation^37^ and around 10–100 times less than for CIML-NK generation^20^. Cytokine-priming alone is sufficient for NK cell activation in vitro, as evidenced by the increase in CD56 on their surface^38^. However, BCG-priming confers features that improve the proliferation capacity of activated CD56^high^ NK cells, as well as effector function and persistence in vitro. Interestingly, no significant difference was described in either tumour cell recognition or specific lysis capacity when briefly stimulating BIL-NK cells with 5 ng/ml IL18 prior to the experiment (Fig. S9).

BIL-NK cells express high levels of NKG2D and show efficient anti-tumour cytotoxic activity. In fact, our functional data demonstrate that tumour killing depends principally on NKG2D, with some contribution from NKp46 and DNAM1. The action of these activating and adhesion receptors likely counteracts the expression of NKG2A, a receptor that associates with CD94 to form heterodimers with inhibitory function^39,40^. NKG2A is currently regarded as a checkpoint receptor that decreases the ability of NK cells to recognize tumour cells, leading to lower cytotoxic function and IFNγ production^41–44^. Nevertheless, our data confirm previous results showing that NKG2D ligation can transduce a dominant stimulatory signal to NK cells, overcoming an MHC class I–mediated inhibitory signal and triggering NK cytotoxicity^45,46^. These observations are also consistent with other reports of anti-tumour activity mediated by NKG2A^+^ NK cells^20,24^. Consistent with the lack of IL18 receptor activation, B-pNK cells do not significantly increase IFNγ production after target cell encounter. The specific lysis capacity was not affected by stimulation with 5 ng/ml IL18 1d prior to the experiment.

Previous research has shown that adaptive-like NK cells from HCMV-seropositive individuals are characterized by the expansion of an NKG2C^+^ subset with a CD56^dim^NKG2A^–^CD57^+^KIRs^+^ phenotype, which has been associated with NK cell maturation^47,48^. Interestingly, the NKG2C^+^ subpopulation found here, after BCG-priming followed by cytokine expansion, was CD56^high^NKG2A^+^CD57^-^FcεRIγ^+^. These cells were still functional and proliferated for several weeks in vitro. Our results are consistent with a proliferation capacity described for FcεRIγ^+^ NK cells^35^. Another intriguing finding is the co-expression of both NKG2A and NKG2C in anti-tumour activated NK cells. Both NKG2A and NKG2C associate with CD94 to form heterodimers with inhibitory and activating function respectively, upon recognition of HLA-E/peptide complexes with different affinities^39,40^. One could speculate that in tumours treated with BCG, the mycobacteria could provide a peptide to HLA-E that would favour binding NKG2C over NKG2A, since receptor affinity depends on peptide identity^40^. In fact, mycobacteria peptides presented by HLA-E to T cells have been described, but the affinity to also bind CD94/NKG2A or C complexes have not been studied^49,50^. In any case, further research may shed light on the integration of signals by these related receptors that could help designing the use of anti-NKG2A based therapies^51^^.^.

The efficacy of intravenous BCG as anti-tumour therapeutic agent has been demonstrated in murine models for bladder and lung cancers^52,53^. Although some studies report that intravenous BCG is not toxic in primates^54^, using whole bacteria systemically could be a concern in cancer patients. For this reason, our data showing that mycobacteria did not grow in cell culture conditions could suggest minimal complications. Further, the protective effect of antibiotic prophylaxis could avoid any potential complications in patients. Moreover, since expansion of anti-tumoral CD56^high^ NK cells can also be achieved be either dead mycobacteria or mycobacteria-derived products^55^, future research will explore the avenues to avoid administration of whole bacteria to immunocompromised cancer patients.

From a translation perspective, the BCG-priming followed by minimal-dose cytokine approach has several advantages when compared to other cytokine-mediated stimulation strategies for ex vivo expansion of NK cells. First, cultures start with PBMC and, without any cell sorting, can result in up to 80% NK cell cultures, simplifying enormously handling time and procedures. Second, the use of minimal doses of each cytokine, together with the weekly frequency provides an important cost-related advantage. Further, the method enhanced cytotoxic function and proliferation rates while maintaining cells in a healthy, activated state, demonstrated by expression of CD25 and CD69. These findings suggest long-term survival of BIL-NK cells, even in resting conditions, a very desirable feature for in vivo translation. Third, this strategy does not need transfection of a cytokine membrane-bound construct for expansion of effector NK cells with anti-tumour phenotype, which requires a method for self- elimination. Since BIL-NK cells could efficiently kill multiple different types of tumours, this could provide a first step towards a novel strategy to develop allogeneic immunotherapeutic cells against solid tumours.

## Materials and methods

### BCG and peripheral blood populations

PBMC from buffy coats of healthy donors and donors with known serology for HCMV were obtained from the Regional Transfusion Centre, Madrid, with informed consent from the participants and with the ethical permission and experimental protocols approved by local and CSIC bioethics committees. All methods were carried out in accordance with biosafety guidelines and regulations authorized by CNB-CSIC.

PBMC were isolated by centrifugation on Ficoll-HyPaque and cultured in complete (2 mM L-glutamine, 0.1 mM nonessential amino acids, 1 mM sodium pyruvate, 100 U/mL penicillin, 100 U/mL streptomycin, 10 mM Hepes, 50 µM β-mercaptoethanol) RPMI-1640 medium (Biowest) supplemented with 5% FBS (Capricorn), 5% HS (Sigma).

BCG-Tice strain (OncoTICE, MSD) (previously calculated as 2% live bacteria^55^) aliquots were reconstituted in RPMI-1640 medium 10% DMSO and stored at − 80 °C. Viability decreased to < 0.1% after several months frozen. For some experiments, live cultures of GFP-expressing BCG Pasteur bacteria were used (GFP-positive bacteria > 90%)^56^. Viable bacteria counting was performed as previously described^55^.

### BCG-mediated stimulation

PBMC co-culture with BCG in vitro model was described previously^23,55^. Briefly, 10^6^ PBMC/ml were incubated in 24-well plates with or without BCG at a 6:1 ratio (total bacteria to PBMC). After one week in culture, cells in suspension were either recovered from the co-culture, centrifuged, and analysed by flow cytometry or kept in culture for another week, as indicated.

### Cell lines

All cell lines were genotyped for authentication at the genomics service of the Instituto de Investigaciones Biomédicas (IIBM-CSIC, Madrid). The bladder cancer cell lines T24, J82, and RT-112 were previously described^23^. Human metastatic melanoma cell lines Ma-Mel-86c and Ma-Mel-86f, provided by Prof. Annette Paschen (University Hospital of Essen, Germany), were described previously^57,58^. The MCF7 and MDA-MB-231 breast adenocarcinoma, the MDA-MB-453 breast metastatic carcinoma, the SW480 colon adenocarcinoma, the MKN45 gastric cancer, the K562 erythroleukaemia cells, the p815 mouse mastocytoma, and the A549 and H3122 lung cancer cell lines were cultured in complete RPMI-1640 medium (Biowest) supplemented with 10% FBS. All cells were kept at 37 °C, with humidified atmosphere of 5% CO_2_. Cells were regularly tested for mycoplasma contamination.

### Flow cytometry

Cells were washed with PBA [PBS supplemented with 0.5% bovine serum albumin (BSA), 1% FBS and 0.065% sodium azide] and incubated with antibodies against surface markers: CD3-FITC, CD3-PB, CD16-PE/Cy7, CD4-PC5.5, CD8-APC/Cy7, CD25-PE/Cy7, CD69-APC/Cy7, CD56-PE, CD57-FITC, LAMP1 (CD107a)-APC; CD158a (KIR2DL1/S1/S3/S5)-PE/Cy7, CD158b/j (KIR2DL2/L3/S2)-PE/Cy7, CD158e1 (KIR3DL1, NKB1)- PE/Cy7, CD279 (PD1)-PE, CD223 (LAG3)-PE, CD314 (NKG2D)-PE/Cy7, CD335 (NKp46)-PE, CD366 (TIM3)-PE, IgG1 isotype control (MOPC21)-PE, TIGIT (VSTM3), and anti-rabbit IgG-BV510 (Biolegend); CD56-PC5 and NKG2A-APC (Beckman Coulter); polyclonal goat anti-mouse immunoglobulin-RPE (Agilent-Dako), IgG2a isotype control (UPC10) and Fc-epsilon RI-gamma (Merck); and NKG2C-PE (R&D). For extracellular staining, cells were directly incubated with the appropriate conjugated antibodies at 4 °C for 30 min in the dark. For intracellular staining, after surface labelling, cells were fixed with 1% p-formaldehyde for 10 min at RT, permeabilized with 0.1% saponin for 10 min at RT. After staining, cells were washed in PBA and analysed using either Gallios, CytoFLEX or CytoFLEX S flow cytometers (Beckman Coulter). Analysis of the experiments was performed using Kaluza software.

### scRNA-seq

Methods followed for the sample preparation, library generation, sequencing and data analysis have been described^32^. Briefly, library pools from BCG-stimulated PBMC were sequenced at 650 pM in paired-end reads on a P3 flow cell using NextSeq 2000 (Illumina) at the Genomics Unit of the Centro Nacional de Investigaciones Cardiovasculares (CNIC, Madrid, Spain). Seurat (v4.0.2) R package was used to subset and merge NK cells from the in-house BCG-primed experiment and the dataset of resting NK cells coming from^33^. For visualization purposes, VlnPlot and DoHeatmap functions from Seurat R package were used, as well as stacked_violin and dotplot functions from Scanpy (v1.9.1) Python toolkit. GOBP enrichment analysis was carried out with Metascape platform (https://metascape.org/) and represented using the ggplot2 (v3.3.6) R package. Reactome pathways were explored at single cell level using the escape (v1.4.1) R package.

R code related to the main scRNA-seq figures can be found at GitHub (https://github.com/algarji/Felgueres_NK_scRNA-seq). scRNA-seq data from BCG-priming experiments are available at Gene Expression Omnibus (GEO): GSE203098. scRNA-seq data from resting NK cells in PBMC^33^ are also available at GEO: GSE149689.

### Cytokine-mediated stimulation

Aliquots of rhIL12, 15 and 21 (Peprotech) and rhIL18 (MBK) cytokines were prepared as indicated by the manufacturer and stored at − 80 °C, so that vials were only thawed once. For minimal-dose cytokine titration experiments, PBMC cultures were stimulated by adding different concentrations of cytokines either individually or in combination. 7 days later, cells were recovered for characterization and functional assays. Different wells were recovered every seven days after cytokine stimulation.

For persistence and phenotype experiments, after a week of co-culture of PBMC and BCG, cells were re-stimulated by adding IL12, 15 and 21 to a final concentration of 0.1 ng/ml, 0.5 ng/ml, and 0.5 ng/ml, respectively. In certain cases (as indicated), IL18 was added the day before the experiment to a final concentration of 5 ng/ml. Functional assays were performed weekly, either after BCG or cytokine stimulation, and phenotype was monitored by flow cytometry.

### Proliferation assays

PBMCs were incubated with 2 μM CellTrace™ Violet stain (Invitrogen) for 20 min at 37 °C 5% CO_2_. Complete RPMI-1640 medium (Biowest) 5% FBS (Capricorn), 5% HS (Sigma) was then added for 5 min and the cells were washed once with complete medium before plating in 24-well plates with minimal-dose IL12, 15 and 21 (0.1 ng/ml, 0.5 ng/ml, and 0.5 ng/ml, respectively). After seven days in culture, cells were recovered and analysed by flow cytometry.

### Degranulation experiments

PBMC from healthy donors were used as effector cells. Cancer cell lines, pre-treated with HP1F7 antibody, which was included in the medium at a final concentration of 10 μg/ml for 30 min, to block MHC-I mediated NK inhibition (unless otherwise indicated), were used as target cells. K562 cells were used as positive control. 10000 effector cells (normalizing for NK cells) were incubated with 20000 target cells (1:2 E:T ratio), unless otherwise indicated, for 2 h as described^59^. Surface expression of LAMP1 (CD107a) was analysed by flow cytometry.

### Cytokine release assays, intracellular staining

PBMC were co-cultured with target cells for 6 h at 1:2 E:T ratio (normalizing for NK cells) at 37 °C, 5% CO_2._ After 1 h of co-incubation, brefeldin-A (Biolegend) was added to a final concentration of 5 μg/ml. 5 h later, cells were recovered, fixed, permeabilised, and stained for intracellular IFNγ, MIP-1β and TNFα, using anti-IFNγ-PE or -APC/Cy7, anti-TNFα-APC (Biolegend) and anti-MIP-1β-PE (BD Pharmingen).

### Cytotoxicity assays

10^4^ target cells were plated in 96-well flat-bottom plates in triplicates in a final volume of 0.2 mL and let to adhere overnight. The next day cells were labelled for 1 h with medium containing 3 µM calcein-AM (Invitrogen), washed 3 times and incubated in fresh medium for a further hour to release free dye. Target cells were then pre-treated with HP1F7 to block MHC-I, unless otherwise indicated. Cells were resuspended in complete RPMI-1640 without phenol red (Gibco) to minimise interference. Effector cells were incubated with adherent target cells for 3 h at 37 °C and 5% CO_2_at a 5:1 E:T ratio (unless otherwise indicated); percentage of NK cells was determined for each donor and cell numbers adjusted accordingly. Supernatants were recovered, after centrifugation at 270 x g for 5 min to pellet cells and transferred to a clean opaque plate. Calcein-AM release was determined by measuring absorbance (excitation wave 485 nm and emission wave 535 nm), using BioNova® F5 System. Specific lysis was calculated as the ratio [(value − spontaneous release)/(maximum release − spontaneous release)] × 100. Spontaneous release corresponds to labelled target cells without effector cells. Maximum release was determined by lysing the target cells in 0.5% Triton X-100 (Invitrogen). In all the experiments, the spontaneous release was between 20 and 30% of the maximum release. For blocking experiments, effector cells were pre-treated for 20 min with 4 μg/ml of purified anti-human NKG2D (Millipore), TRAIL (Biolegend), FASL (Biolegend), NKp46 (R&D Systems), NKG2A (Beckman Coulter), DNAM-1 and NKG2C (R&D Systems), where murine IgG1 and IgG2b (Invitrogen) were used as isotype controls.

### Patient samples

The experiments were conducted with the understanding and written informed consent of each participant, or their legal tutor, and approved by local and regional ethical committees and conformed to the principles set out in the WMA Declaration of Helsinki and the Department of Health and Human Services Belmont Report. Blood samples from two NMIBC (T1G3) patients (P25: age 62, male; and P26: age 67, male) enrolled in a prospective study between 2015 and 2016 at Hospital Infanta Sofía (Madrid, Spain) (Institutional Review Boards, numbers and dates: CEI La Paz Hospital HULP-1067 Feb 8th 2011; revised by CEI Infanta Sofía Hospital and CSIC Local Ethical Committee; extension HULP-2338, April 25th 2016) as described in^60^ were used. Also, remnants of BM samples from a CNS myeloid sarcoma (P1: age 12, female) and two common B cell acute lymphoblastic leukaemia (B-ALL) paediatric patients (P2: age 7, female, 84% blast cells; P3: age 11, female, 75% blast cells), obtained for diagnosis, were approved for their use in research, according to the guidelines of La Paz Hospital local Ethics Committee (approval: PI-3398). Cells were cryopreserved in FBS containing 10% DMSO at − 80 °C in aliquots until analysis.

### Statistics

Graphpad Prism 9 software was used for statistical analysis and representation of the data. Results were presented either as individual values or as the mean and standard deviation (SD) (mean ± SD), as indicated in the figure legends. A two-tailed, paired, parametric t-test was used to compare variations of the same group of healthy donors with time. Statistically significant differences were evaluated according to the following criteria: * *p* < 0.05, ** *p* < 0.01, * *p* < 0.001, **** *p* < 0.001 (α = 0.05). When *p* > 0.05, differences were considered not significant (ns).

## Data Availability

Data were generated by the authors and are available on request. R code related to the main scRNA-seq figures can be found at GitHub (https://github.com/algarji/Felgueres_NK_scRNA-seq). scRNA-seq data from BCG-priming experiments are available at Gene Expression Omnibus (GEO): GSE203098. scRNA-seq data from resting NK cells in PBMC (38) are available at GEO: GSE149689. The corresponding author, Mar Valés-Gómez (mvales@cnb.csic.es), should be contacted if someone wants to request the data from this study.

## Supplementary Figures

Supplementary Figures.
