# Supplementary material for: BCG priming followed by a novel interleukin combination activates Natural Killer cells to selectively proliferate and become anti-tumour long-lived effectors
Source: Sci Rep. 2024 Jun 7;14:13133. doi: 10.1038/s41598-024-62968-2 (PMC11161620; doi:10.1038/s41598-024-62968-2)
Supplement: Supplementary file 1 — Supplementary Figures. [file 41598_2024_62968_MOESM1_ESM.pdf]

BCG priming followed by a novel interleukin combination activates Natural Killer cells to selectively proliferate and become anti-tumour long-lived effectors

María-José Felgueres[1], Gloria Esteso[1], Álvaro F. García-Jiménez[1], Ana Dopazo[2,3], Nacho Aguiló[4], Carmen Mestre-Durán[5,6], Luis Martínez-Piñeiro[7], Antonio Pérez-Martínez[5,6,8], Hugh T. Reyburn[1], and Mar Valés-Gómez[1*]

SUPPLEMENTARY INFORMATION

[1] Department of Immunology and Oncology, National Centre for Biotechnology, Spanish National Research Council (CNB-CSIC), Madrid, Spain.
[2] Genomics Unit, Centro Nacional de Investigaciones Cardiovasculares (CNIC), Madrid, Spain.
[3] CIBER de Enfermedades Cardiovasculares (CIBERCV), Madrid, Spain.
[4] Department of Microbiology, Pediatrics, Radiology and Public Health of the University of Zaragoza, IIS Aragon, CIBER de Enfermedades Respiratorias, Spain.
[5] Translational Research in Pediatric Oncology, Hematopoietic Transplantation and Cell Therapy, IdiPAZ, and Pediatric Hemato-Oncology, Hospital Universitario La Paz, Madrid, Spain.
[6] IdiPAZ-CNIO Pediatric Onco-Hematology Clinical Research Unit, Spanish National Cancer Research Centre (CNIO), 28049 Madrid, Spain.
[7] Urology Department and Hospital La Paz Institute for Health Research (IdiPAZ), La Paz University Hospital, Madrid, Spain.
[8] Pediatric Department, Autonomous University of Madrid, Madrid, Spain.
[*] Corresponding author

Fig S1.

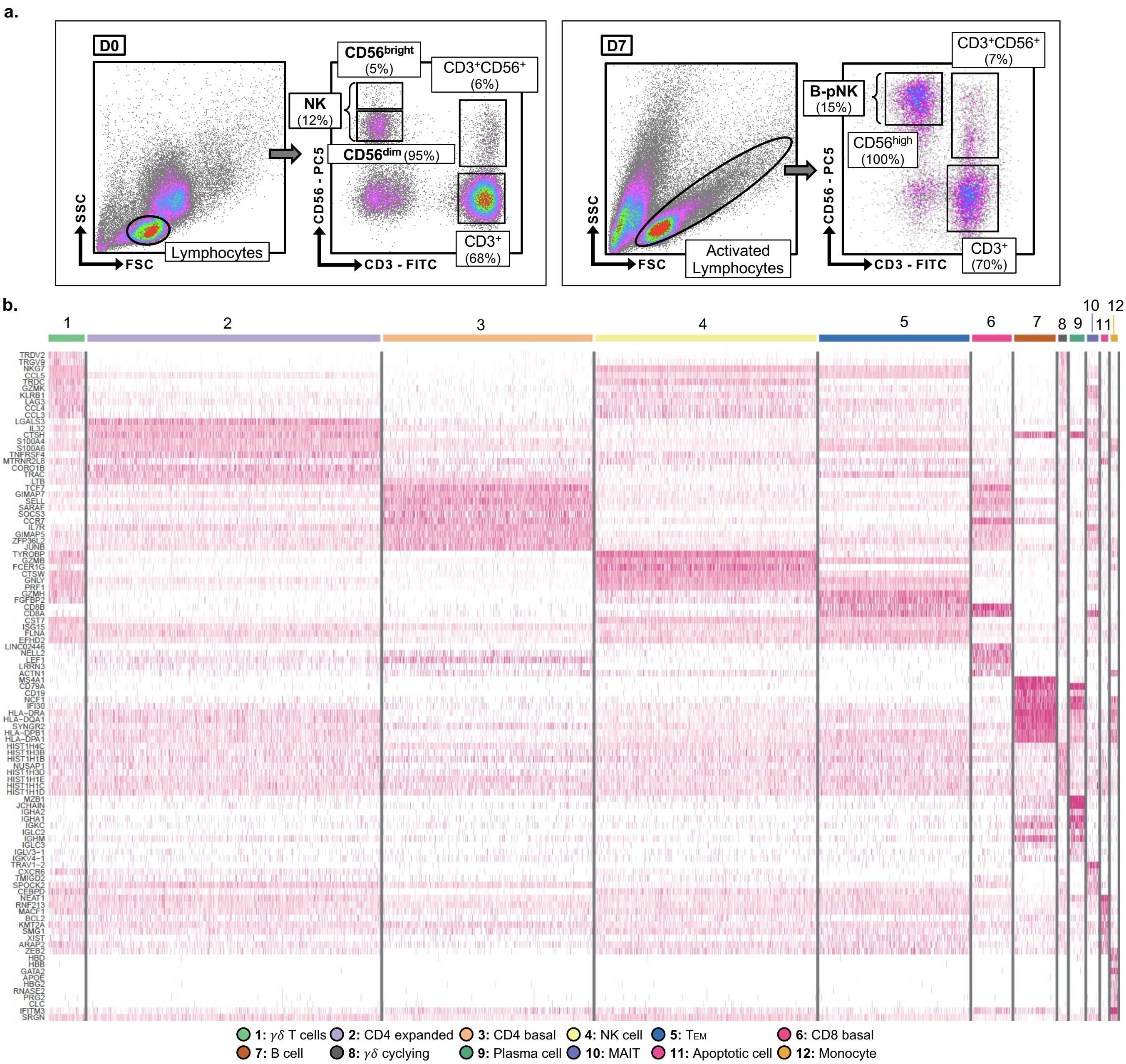

Fig. S1. B-pNK characterization. a. Gating strategies. Freshly thawed PBMC were analysed by flow cytometry. NK cells, $CD3^+$ lymphocytes and double positive $CD3^+CD56^+$ cells were selected, as indicated, within the lymphocyte region in FSC vs SSC. At day 7, BCG-activated lymphocytes were selected in the FSC vs SSC plot. Activated $CD56^{high}$ NK cells were gated separately from the rest of $CD3^-CD56^+$ NK cells. **b. Differential gene expression.** Heat-map of the whole-data set representing a scaled expression of the top 10 differentially expressed genes across the 12 clusters identified in BCG-activated PBMC from 3 healthy donors recovered from the co-culture at day 7.

a.

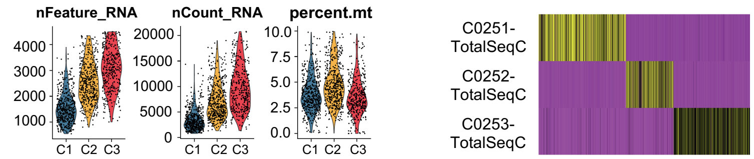

Demultiplexing

	Sample 1	Sample 2	Sample 3	TOTAL
C1	255	146	80	481
C2	155	75	235	465
C3	131	74	157	362
TOTAL	541	295	472	1308

b.

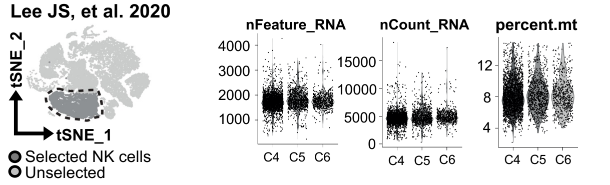

Demultiplexing

	HD1	HD2	HD3	HD4	TOTAL
C4	392	642	336	1088	1458
C5	296	244	223	106	870
C6	308	223	147	2	680
TOTAL	996	1109	706	1196	4007

c.

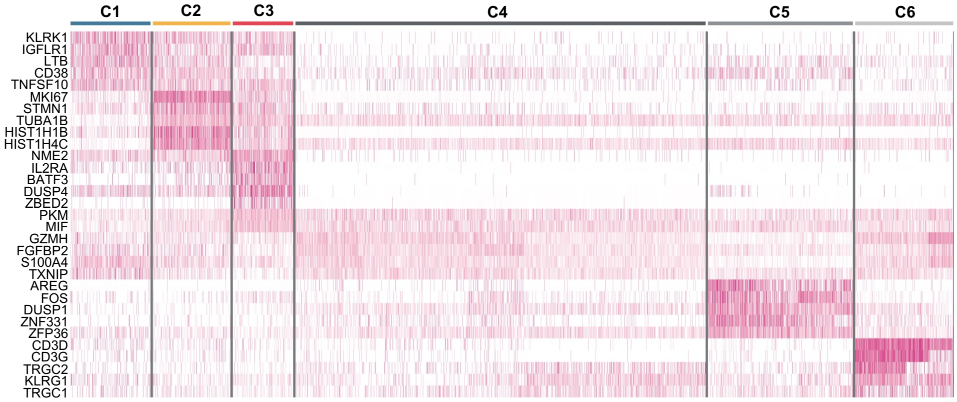

d.

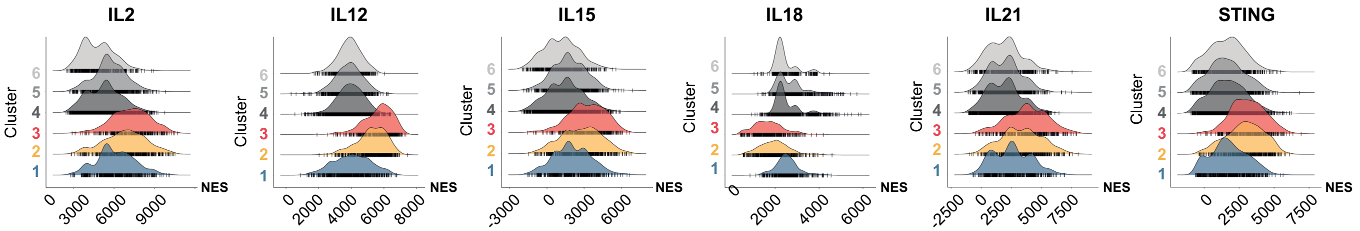

e.

**IL2**	*IL2RB,JAK3,IL2,PTK2B,STAT5A,IL2RA,IL2RG,SHC1,JAK1,SYK,STAT5B,LCK*
**IL12**	*RALA,CNN2,CDC42,IL12RB2,RPLP0,JAK2,IL12RB1,MTAP,PSME2,TYK2,AIP,IFNG,,HSPA9,IL12B,LMNB1,CAPZA1,TCP1,HNRNPA2B1,VAMP7,RAP1B,SNRPA1,CA1,LCP1,IL10,STAT4,SOD1,ARF1,MSN,GSTO1,PDCD4,HNRNPDL,JAK1,IL12A,BOLA2B,HNRNPF,CFL1,PITPNA,TALDO1,PAK2,ANXA2,BOLA2,P4HB,PPIA,SERPINB2,MIF,GSTA2,MIF,PSME2,SOD2*
**IL15**	*GAB2,IL2RB,SOS2,JAK3,SOS1,STAT5A,IL15RA,IL2RG,SHC1,JAK1,IL15,STAT3,STAT5B,GRB2*
**IL18**	*ALOX5,IL4,IL18R1,IL18RAP,IL37,IL18BP,IL18,IL13,ALOX5*
**IL21**	*IL21R,JAK3,STAT1,STAT5A,STAT4,IL21,IL2RG,JAK1,STAT3,STAT5B*
**STING**	*MRE11,XRCC5,DTX4,IRF3,TRIM21,NLRP4,IFI16,CGAS,STAT6,NLRC3,DDX41,TBK1,STING1,XRCC6,TREX1,PRKDC,STING1*

Fig. S2. In depth analysis of BCG-primed and peripheral blood NK cells. a, b. scRNA-seq quality control of B-pNK and freshly isolated NK dataset. NK cells from BCG-activated PBMC from 3 healthy donors (a) and resting PBMC NK cells from 4 healthy donors (Lee et al. Sci. Immunol. 2020. DOI: 10.1126/sciimmunol.abd1554) (b) were analysed by scRNA-seq. Cells were filtered to retain between 200 and 5000 features, and present less than 10% of mitochondrial content. Doublets and negative cells were additionally removed based on the detection of HTO signal (middle). Tables summarize the number of events per sample and cluster. **c. Differential gene expression comparing B-pNK and freshly isolated NK cells.** Heat-map of the whole-data set representing a scaled expression of the top 5 differentially expressed genes across the different NK clusters from BCG-primed NK cells (C1, C3, C3) and PBMC NK cells (C4, C5, C6). **d. Selected reactome pathways.** Histograms showing the normalized enrichment scores (NES) of the differentially expressed genes involved in interleukin (IL) 2, 12, 15, 18, and 21 and cGAS-STING reactome pathways for both B-pNK clusters (1 - 3) and PBMC NK cell clusters (4 – 6). **e. Genes within selected pathways**. Histograms in (d) were built after analysing the genes included in (IL) 2, 12, 15, 18, and 21 and cGAS-STING pathways within the pathways within the Reactome Pathway Database (https://reactome.org/).

Fig S3.

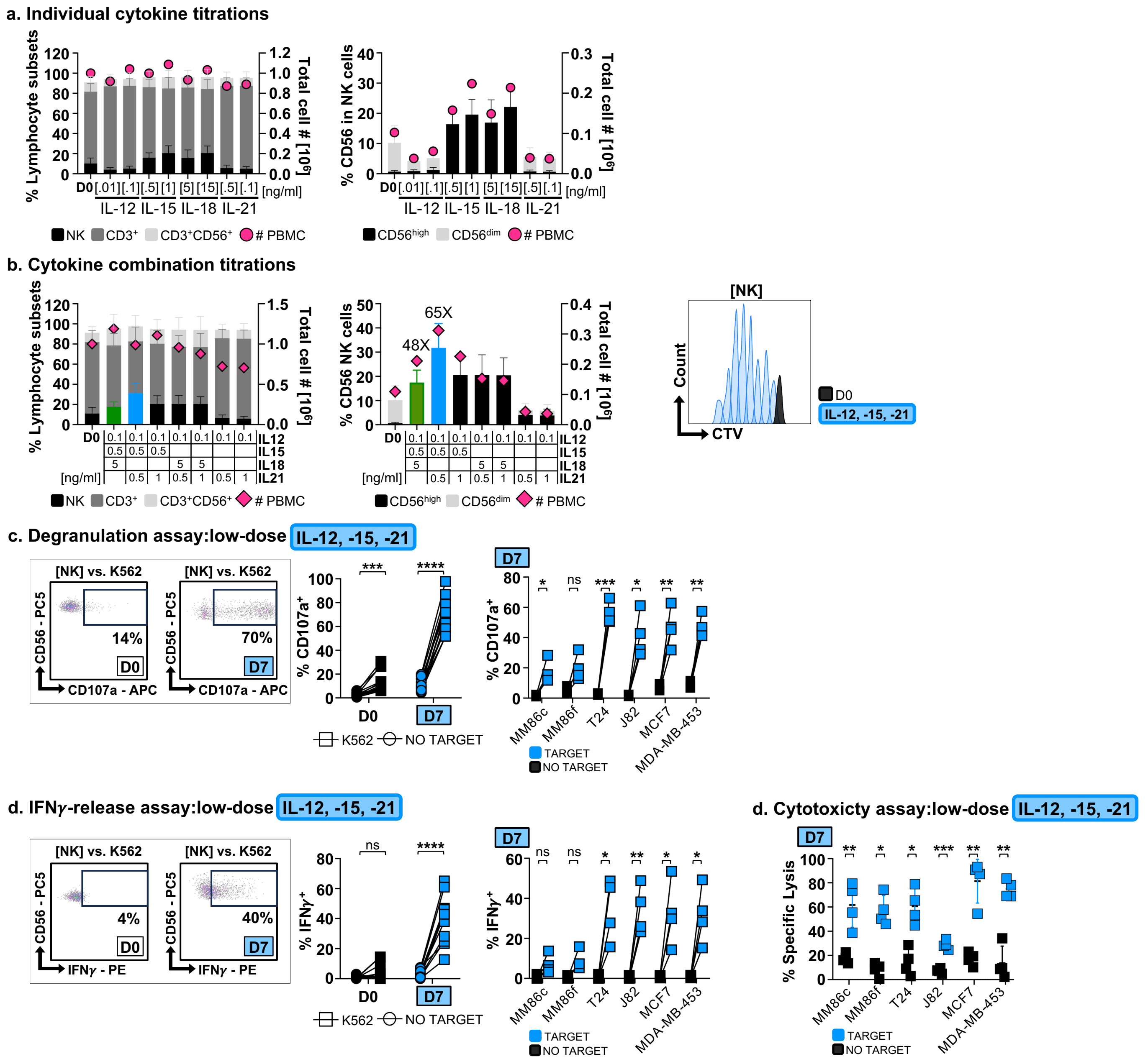

Fig. S3. Minimal doses of IL12, 15, and 21 enhance proliferation and function of anti-tumour NK cells *in vitro*. a. Effect of low-dose cytokines in lymphocyte expansion. PBMC from 10 healthy donors were incubated with minimal doses of cytokines as indicated (X axis). After a week in culture, cells were counted (●, right Y axis) and analysed by flow cytometry (left). The % and SD of the different lymphocyte subsets are depicted with different shadows (left Y axis). CD56 upregulation in the activated NK cell subset is represented in a sperate plot (right). **b. Effect of low-dose cytokines in combination.** PBMC from 10 healthy donors were incubated with minimal doses of cytokines in combinations, as indicated (X axis). After a week in culture, cells were counted (◆, right Y axis) and analysed by flow cytometry. The two cytokine combinations which triggered proliferation and CD56 upregulation the most are highlighted in green and blue, as colour code for other experiments. A representative proliferation plot of the PBMC culture co-incubated with the IL12, 15 and 21 cytokine combination is shown. **c-e. Degranulation, IFNγ-release, and cytotoxicity assays.** After cytokine activation with the selected combination, 25000 effector NK cells (1:2 E:T ratio) from 4 healthy donors were tested against solid tumour target cell lines: melanoma (MM86c, MM86f), bladder (T24, J82); and breast (MCF7, MDA-MB-453) cancers for degranulation (c) and IFNγ - release (d). K562 cells were used as positive control (n=12). Surface LAMP-1 (CD107a) (c) and intracellular IFNγ (d) were measured by flow cytometry. Representative dot plots of degranulation and IFNγ release against K562 cells are shown. For cytotoxicity assays (e), effector NK cells were incubated with solid tumour target cells labelled with calcein-AM (5:1 E:T ratio, NK to target). Dye-release was measured in 3-hour experiments and specific lysis was calculated as % of spontaneous release. Results against solid tumour targets were obtained in 2 independent experiments. Statistical analyses were done by paired sample t-tests (*p <0.05, **p <0.01, ***p <0.001, ****p <0.0001).

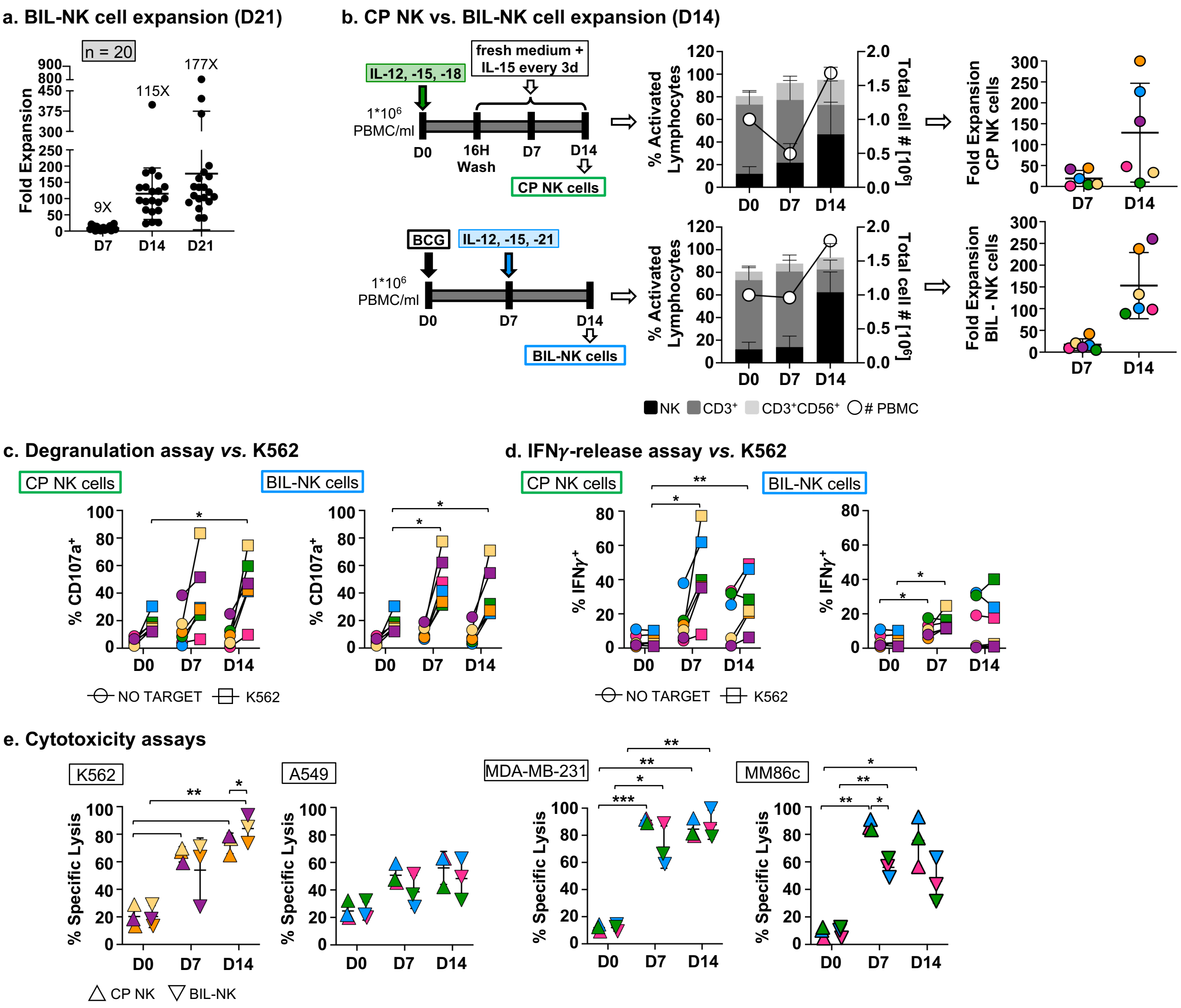

Fig. S4. BIL-NK show higher proliferation capacity and similar effector function against IL-12, -15, and -18 than cytokine-primed (CP) NK cells. a. B-pNK cell expansion with minimal-dose cytokines. BCG-primed PBMC cultures were stimulated after a week in culture with minimal-dose IL12, 15, and 21 and then weekly up to 21 days (n=20, 8 independent experiments). Effector CD56^{high} NK cell fold expansion value for each donor was calculated as described in main text and represented in a scatter plot (mean ± SD error bars). **b. Expansion after 14 days.** For CP NK cells, PBMC from 6 healthy donors were co-cultured with IL12 [10 ng/ml], 15 [1 ng/ml], and 18 [50 ng/ml], for 16 hours. PBMC were then washed 3 times and left in culture in medium with 1 ng/ml IL15, as previously described (Romee et al Blood 2012. DOI: 10.1182/blood-2012-04-419283). BIL-NK cells were obtained after incubating PBMC, from the same 6 healthy donors, with BCG for a week and then stimulated once with IL12 [0.1 ng/ml], 15 [0.5 ng/ml], and 21 [0.5 ng/ml] and incubated another week in culture. Cells were counted (○, right Y axis) and analysed by flow cytometry. The % and SD of activated lymphocytes subsets (left Y axis) are shown in different shades, as indicated. Expansion of effector NK cells was calculated as described previously. Results were obtained in 2 independent experiments. **c-e. Degranulation, IFNγ-release, and cytotoxicity assays.** CP NK and BIL-NK cells were tested as effector cells in 2 independent experiments. For degranulation (c) and IFNγ-release (d), 25000 effector NK cells (1:2 E:T ratio) from 6 donors were tested against K562 cell line. Surface LAMP-1 (CD107a) and intracellular IFNγ were measured by flow cytometry. For cytotoxicity assays (e), effector NK cells from 3 healthy donors were incubated with target cells labelled with calcein-AM (30:1 E:T ratio, total PBMC to target). Target cell lines were K562 erythroleukaemia cells and solid tumour cell lines from lung (A549), breast (MDA-MB-231), and melanoma (MM86c) cancers. Statistical analyses were done by paired sample t-tests (*p <0.05, **p <0.01, ***p <0.001, ****p <0.0001).

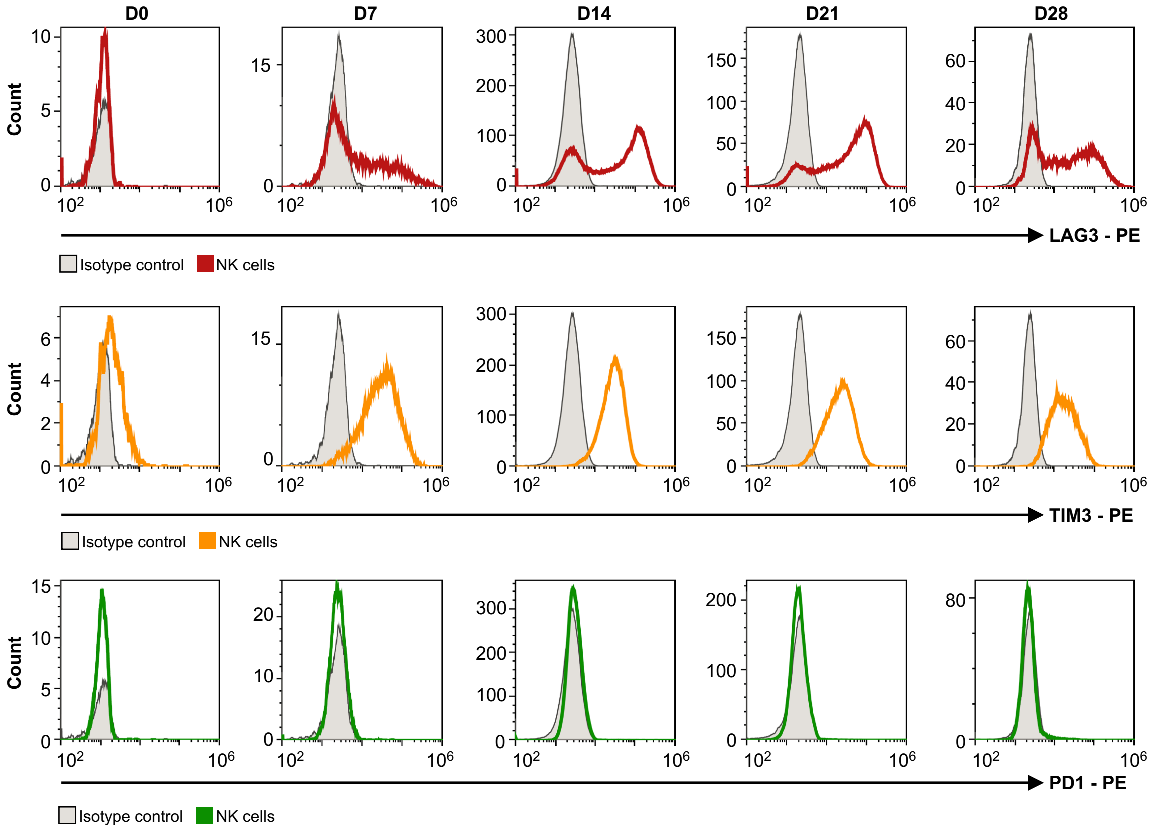

Fig. S5. Expression of immune checkpoints on BIL-NK cells after four weeks in culture. Representative histograms show the expression profile for LAG3 (red), TIM3 (orange) and PD1 (green) exhaustion markers within the NK subset against the IgG isotype control (grey) for each time point (day 0, 7, 14, 21, and 28).

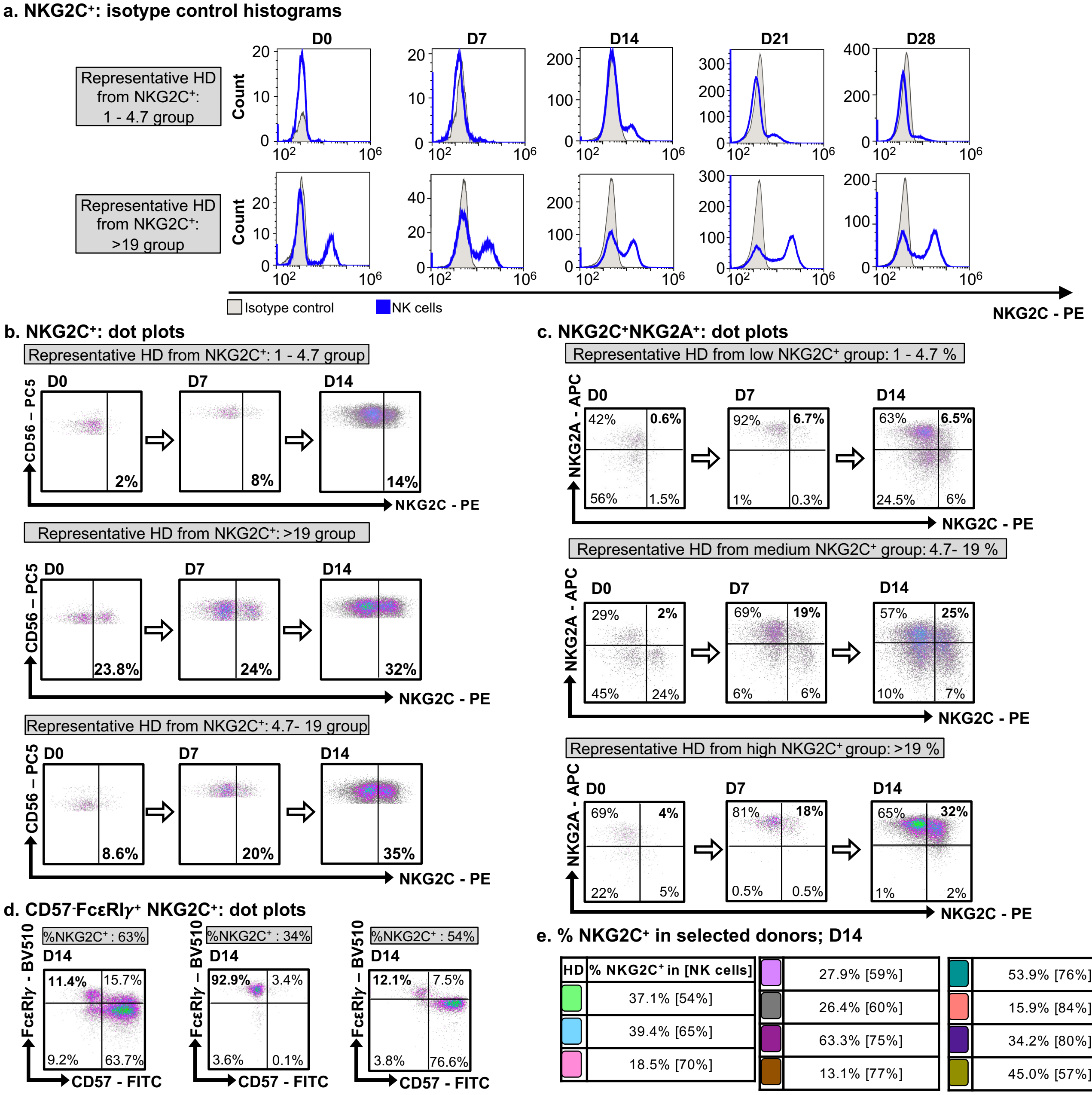

HD	% NKG2C⁺ in [NK cells]	HD	% NKG2C⁺ in [NK cells]	HD	% NKG2C⁺ in [NK cells]
	37.1% [54%]		27.9% [59%]		53.9% [76%]
	39.4% [65%]		26.4% [60%]		15.9% [84%]
	18.5% [70%]		63.3% [75%]		34.2% [80%]
			13.1% [77%]		45.0% [57%]

Fig. S6. Characterization of NKG2C⁺ BIL-NK subsets. a-d. Flow cytometry plots. Representative histograms from 2 healthy donors (corresponding to Fig. 6, main text) with either low (upper) or high (lower) NKG2C basal expression show the expression profile of this marker (blue) within the NK subset against the IgG isotype control (grey) for each time point (day 0, 7, 14, 21, and 28) (a). Representative dot plots from three healthy donors (corresponding to Fig. 6, main text), each with a different basal NGK2C expression, are shown. NKG2C⁺ BIL-NK cells were selected in a NKG2C vs CD56 plot within the total NK cell gate (b). NKG2C⁺NKG2A⁺ NK cells were selected in a NKG2C vs NKG2A plot within the total NK cell gate (c). CD57⁻FcεRIγ⁺ and CD57⁺FcεRIγ⁻ subsets were selected in a CD57 vs FcεRIγ plot from within the NKG2C⁺ NK region (d). % of each subset are indicated. **e. % NKG2C⁺ NK cells and total NK cells of selected donors after 2 weeks in culture.** Table shows the % of the NKG2C⁺ subset within the BIL-NK population for each donor used in the functional assays depicted in Fig. 6 (colour coding is maintained). The total % of effector BIL-NK cells for each donor, selected within the activated lymphocyte population, is specified within brackets.

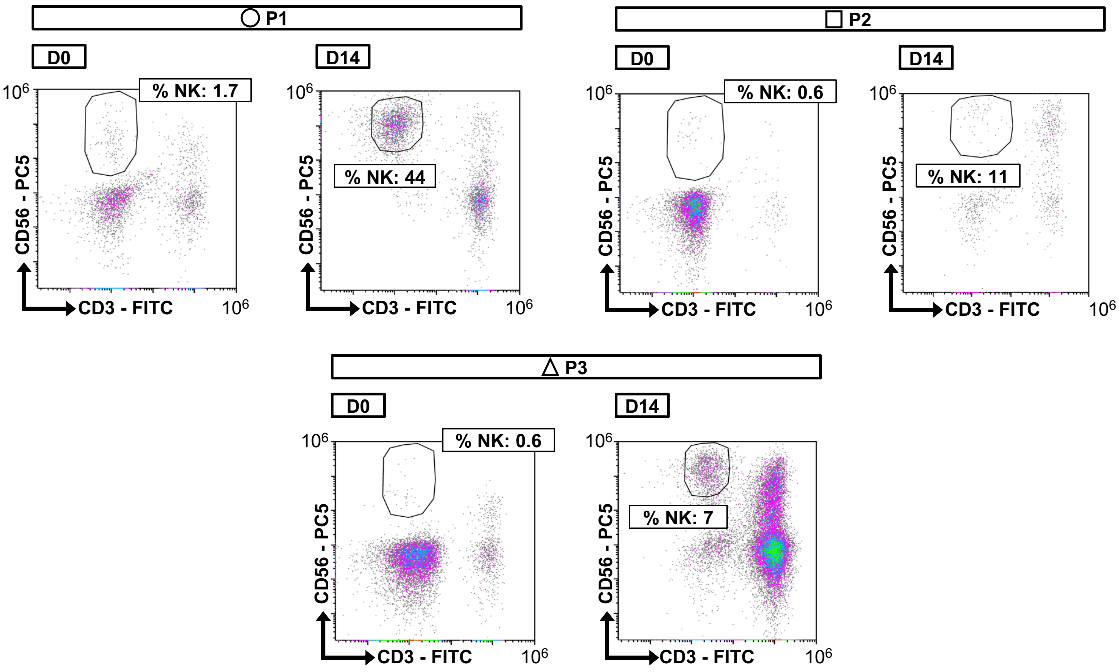

Fig. S7. Generation of BIL-NK from paediatric cancer patients. BMMC from one CNS myeloid sarcoma paediatric patient (P1) and two B-ALL paediatric patients (P2, P3) were incubated with BCG and stimulated after a week in culture with minimal-dose IL12, 15, and 21 and then, cells were recovered and analysed via flow cytometry. Basal NK population (D0) and the expanded anti-tumour BIL-NK subset (D14) were determined in CD3 vs CD56 plots. Percentages of NK cells are indicated for each donor.

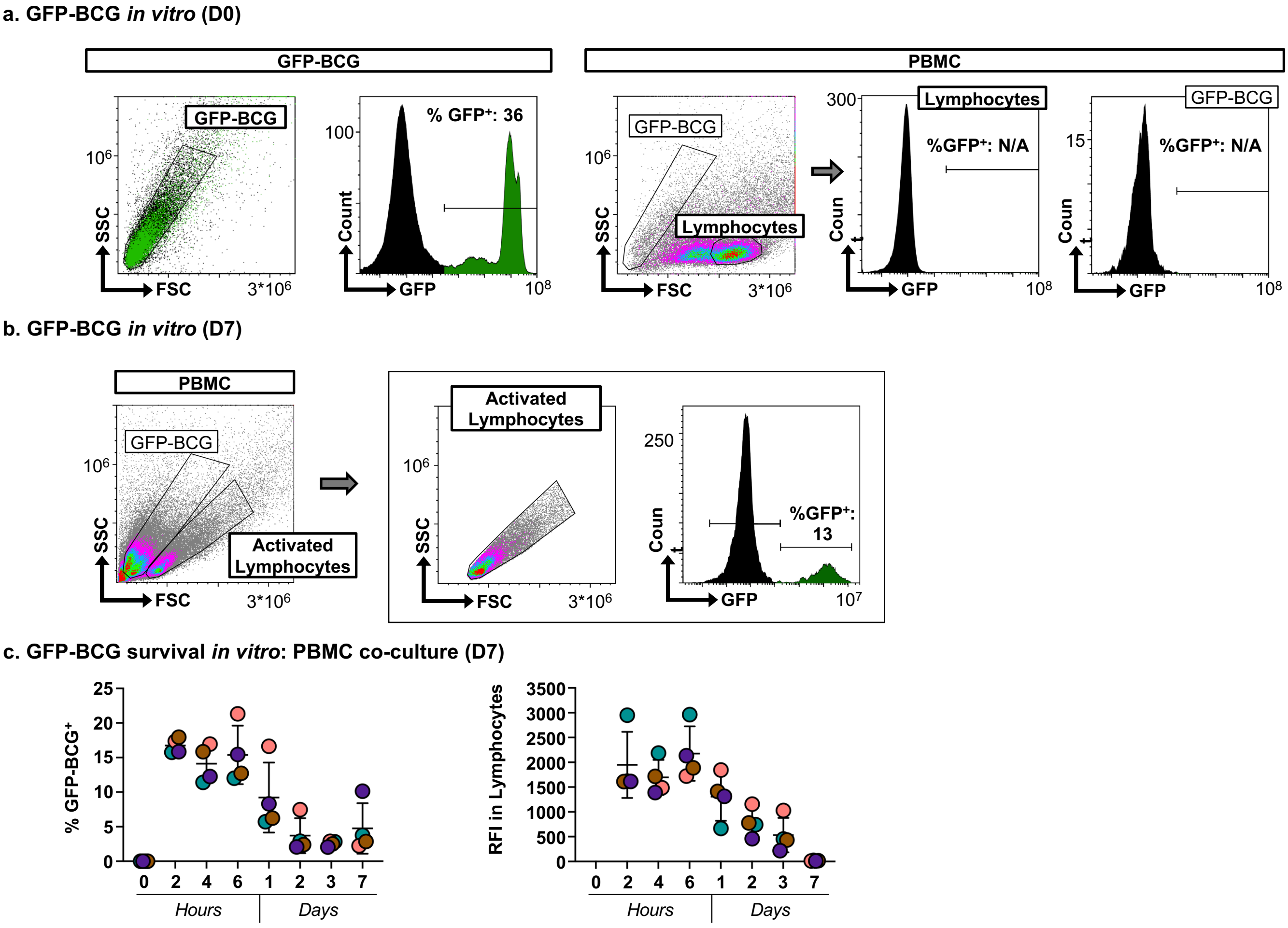

Fig. S8. Live BCG survival *in vitro*. a. Gating strategy. Representative plots show live GFP-BCG (left) and freshly thawed PBMC (right) analysed by flow cytometry to determine BCG vs cell gates and basal intensity of GFP. Although more than 90% BCG should be GFP$^+$, flow cytometry does not separate bacteria from culture debris. So, only 36% GFP$^+$ events were counted. **b. PBMC co-cultured with live GFP-BCG for 7 days.** Activated lymphocytes were selected from FSC vs. SSC plots and the % of GFP$^+$ cells were obtained (right: representative histogram). These cells could have BCG extracellular or intracellular. **c. GFP in PBMC co-cultures.** 10^6 PBMC from 4 healthy donors were co-cultured with $6{\cdot}10^6$ live GFP-BCG. Cells were recovered at the indicated time points (X axis) and analysed by flow cytometry. The % of GFP-BCG present in the activated lymphocyte gate (left) and relative fluorescence intensity [(RFI): RFI=MFI GFP$^+$ / MFI GFP$^-$, where MFI is mean fluorescence intensity] (right) are plotted for each donor (different colours) in a scatter plot (mean ± SD error bars).

Fig S9.

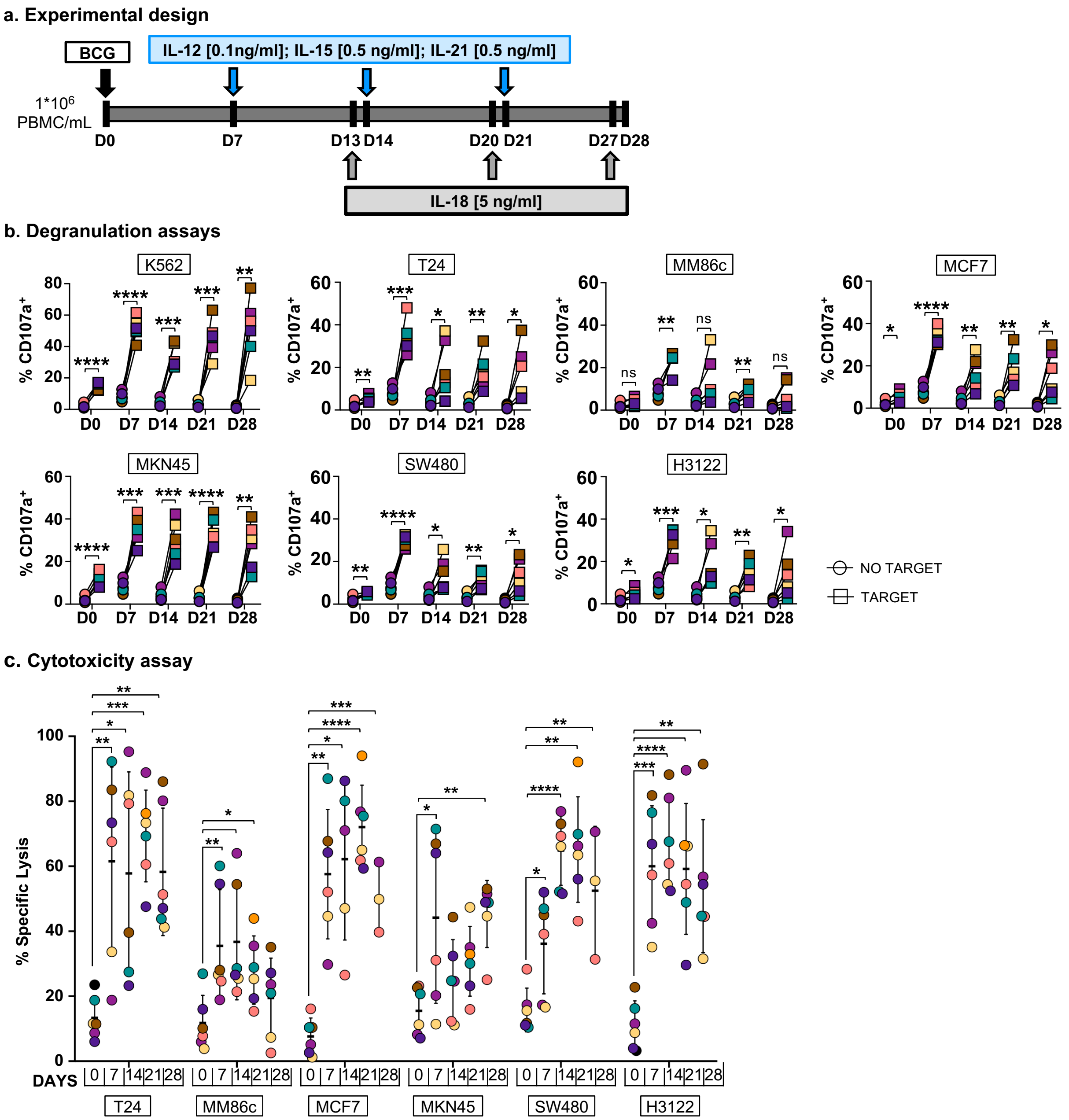

Fig. S9. Effect of low-dose IL-18 boost on effector NK cells one day before functional assays. a. Experimental design. PBMC from 6 healthy donors were incubated with BCG and stimulated with minimal-dose IL12, 15, and 21 after weekly resting periods, as indicated. The day before functional assays, cells were stimulated overnight with IL18 (5 ng/ml). **b, c. Degranulation and cytotoxicity assays.** For degranulation, 10000 NK cells were tested as effector cells against solid tumour target cell lines (1:2 E:T ratio, NK to target): bladder (T24) melanoma (MM86c), breast (MCF7), gastric (MKN45), colon (SW480), and lung (H3122) cancers. K562 was used as positive control. Surface LAMP-1 (CD107a) (b) was measured by flow cytometry. Results were obtained in 2 independent experiments. For cytotoxicity assays (c), effector cells were incubated with solid tumour target cells labelled with calcein-AM (5:1 E:T ratio). Dye-release was measured in 3-hour experiments and specific lysis was calculated as % of spontaneous release. Statistical analyses were done by paired sample t-tests (*p <0.05, **p <0.01, ***p <0.001, ****p <0.0001). Different donors are represented by different colours.
